# The Neural Basis of Avoidance Learning and Its Relation to Safety Processing

**DOI:** 10.1523/ENEURO.0170-26.2026

**Published:** 2026-08-25

**Authors:** Lieselotte Claes, Patrick A. F. Laing, Trevor Steward, Kim L. Felmingham, Bram Vervliet, Christopher G. Davey, Bradford Moffat, Rebecca K. Glarin, Ben J. Harrison

**Affiliations:** ^1^Department of Psychiatry, The University of Melbourne, Parkville, Victoria 3010, Australia; ^2^Laboratory of Biological Psychology, Faculty of Psychology and Educational Sciences, KU Leuven, Leuven 3000, Belgium; ^3^Department of Psychiatry and Behavioural Sciences, University of Texas, Austin, Texas 78712; ^4^Melbourne School of Psychological Sciences, The University of Melbourne, Parkville, Victoria 3010, Australia; ^5^Leuven Brain Institute, KU Leuven, Leuven 3000, Belgium; ^6^The Melbourne Brain Centre Imaging Unit, Department of Radiology, The University of Melbourne, Parkville, Victoria 3010, Australia

**Keywords:** avoidance learning, fMRI, safety, striatum, vmPFC

## Abstract

Persistent avoidance plays a major role in maintaining anxiety disorders. The few studies that have explored the neural basis of avoidance have predominantly examined established avoidance behaviors rather than the learning processes that establish avoidance responses. We aimed to characterize the temporal dynamics and neural mechanisms of avoidance learning using a novel paradigm combined with 7 tesla fMRI in 82 healthy participants (37 male and 45 female). Participants were first conditioned to two threat stimuli and a compound safety stimulus. Next, they were trained to avoid one threat stimulus while the other remained unavoidable. Behavioral data confirmed accurate learning of task contingencies. Successful avoidance was associated with prominent activation of the ventromedial prefrontal cortex (vmPFC), posterior cingulate cortex (PCC), and ventrolateral prefrontal cortex compared with unsuccessful avoidance, with additional nucleus accumbens and orbitofrontal cortex engagement when compared with safety learning (*P*_FDR_ whole-brain corrected). Critically, early compared with late avoidance learning was distinctly associated with activation of midbrain-striatal circuits, including the periaqueductal gray (PAG), mediodorsal thalamus, substantia nigra, and anterior insular cortex—regions associated with threat processing and learning-related signaling. These findings suggest that initial avoidance learning relies on subcortical threat-responsive and learning-related circuits, whereas established avoidance responses engage cortical safety-processing regions. This dissociation parallels recent findings in safety learning and provides novel mechanistic insights for interventions targeting maladaptive avoidance patterns.

## Significance Statement

Avoidance behavior is central to anxiety disorders, yet the neural mechanisms through which avoidance is learned remain poorly understood. Using 7 tesla fMRI and a novel paradigm in 82 participants, we show that initial human avoidance learning engages midbrain-striatal circuits—including the periaqueductal gray, substantia nigra, and thalamus—while established avoidance relies on cortical safety-processing regions centered on the ventromedial prefrontal cortex. This temporal dissociation parallels recent findings in pavlovian safety learning and identifies a potential mechanistic window during which maladaptive avoidance patterns may be most amenable to intervention.

## Introduction

Avoidance behaviors serve to minimize harm from potential danger. Continual reliance on avoidance can be reinforced by the pleasant, relieving sensations evoked by successful threat evasion (Vervliet et al., 2017; Leng et al., 2024). Excessive avoidance is recognized as a key mechanism in the maintenance of anxiety and stress-related disorders, such as posttraumatic stress disorder (PTSD), social anxiety, and panic disorder (Craske et al., 2009; American Psychiatric Association, 2013). Avoidance behaviors also interfere with the efficacy of psychological treatments and can prevent treatment-seeking in the first place (Davies et al., 2015; Brown et al., 2023). Despite the central role of avoidance in anxiety disorders, its underlying neural mechanisms remain unclear, hindering the development of targeted interventions designed to reduce avoidance behaviors.

While several neuroimaging studies have investigated threat avoidance behavior in humans (Delgado et al., 2009; Schlund et al., 2010; Levita et al., 2012; Collins et al., 2014; Boeke et al., 2017; Wendt et al., 2017), few have examined the neural mechanisms supporting initial avoidance learning, a critical gap, as early avoidance learning may represent the period when maladaptive patterns are most modifiable. Previous studies have mostly neglected learning-related processes of avoidance learning by either instructing which stimuli to avoid and how (Wendt et al., 2017) or by training participants before scanning (Schlund et al., 2010; Levita et al., 2012). Other existing fMRI studies have predominantly focused on well-established avoidance responses, reporting consistent activation of the dorsal and ventral striatum, with later learning stages also implicating the ventromedial prefrontal cortex (vmPFC; Delgado et al., 2009; Boeke et al., 2017). However, these studies provide limited insight into the mechanisms driving initial avoidance learning—specifically, how threat expectations are reconciled with safety outcomes as avoidance associations form.

Recent studies distinguish between neural mechanisms that (1) drive initial safety learning and (2) those supporting the behavioral expression of safety at later stages (Laing et al., 2022a). Specifically, studies of fear extinction and conditioned inhibition have identified a prominent role for dopaminergic midbrain-striatal circuits during the early stages of safety learning, consistent with prediction error signaling mechanisms that compute mismatches between expected threat and actual safety outcomes (Hollerman and Schultz, 1998; Gerlicher et al., 2018; Kalisch et al., 2019; Esser et al., 2021; Laing et al., 2022b). For instance, corticostriatal activity is elevated when expectations are violated by threat omission, showing characteristic decreases as safety contingencies become learned (Thiele et al., 2021; Laing et al., 2022b). These findings diverge from the broader neuroimaging literature on safety learning, which has more consistently implicated cortical regions—particularly the vmPFC, hippocampus, and posterior cingulate cortex (PCC)—in the processing of established safety signals (Fullana et al., 2016; Harrison et al., 2017). Rather than encoding new stimulus-safety associations, these cortical regions appear to support the appraisal, affective valuation, and expression of already-learned safety information. Although animal models provide convergent evidence for a critical role of midbrain-striatal prediction error circuitry in driving avoidance learning (Dombrowski et al., 2013; Stelly et al., 2019), the temporal dynamics and circuit-level dissociation between learning-related and expression-related mechanisms (Laing et al., 2022a) have not been directly examined in human studies.

To address this knowledge gap, and to extend our recent work on safety learning dynamics (Laing et al., 2022b), we developed a novel avoidance learning paradigm and combined it with 7 tesla fMRI. First, we examined the brain mechanisms of successful (threat prevented by avoidance response) versus unsuccessful (threat occurs despite avoidance response) avoidance, characterizing changes in brain activity across early and late learning stages. Second, we aimed to compare successful avoidance learning with safety learning to examine whether these processes share common neural substrates (Laing et al., 2022b). Based on evidence from pavlovian safety learning, we hypothesized that early avoidance trials would engage midbrain-striatal circuits associated with stimulus-safety association learning, while late trials would recruit cortical regions associated with safety expression—reflecting the transition from active learning to established avoidance. Finally, we expected that successful avoidance would evoke greater vmPFC activation than pavlovian safety signals, consistent with the enhanced relief and positive affect associated with active avoidance (Vervliet et al., 2017; Leng et al., 2024).

## Materials and Methods

### Participants

Ninety-four participants were recruited for the study via online advertisements. All participants met the following inclusion criteria: (1) aged between 18 and 40 years; (2) no past or current mental health disorder as screened using the Mini International Neuropsychiatric Interview (v7.0.2 for DSM-5); (3) fluency in verbal and written English; (4) no MRI contraindications (e.g., incompatible metal implants, pregnancy, claustrophobia, neurological disorder, traumatic brain injury); and (5) no major hearing or vision impairments. Participants provided written consent and attended a single testing session at the Melbourne Brain Centre Imaging Unit (MBCIU, The University of Melbourne). The study was approved by The University of Melbourne Human Research Ethics Committee.

Twelve participants were excluded from the study due to incomplete or missing data (*n* = 3), technical issues (*n* = 6), or excessive head motion during scanning (*n* = 3, see further). The final sample included 82 participants (45 female) with a mean age of 22.68 (±4.17) years.

### Paradigm

**Figure 1.**
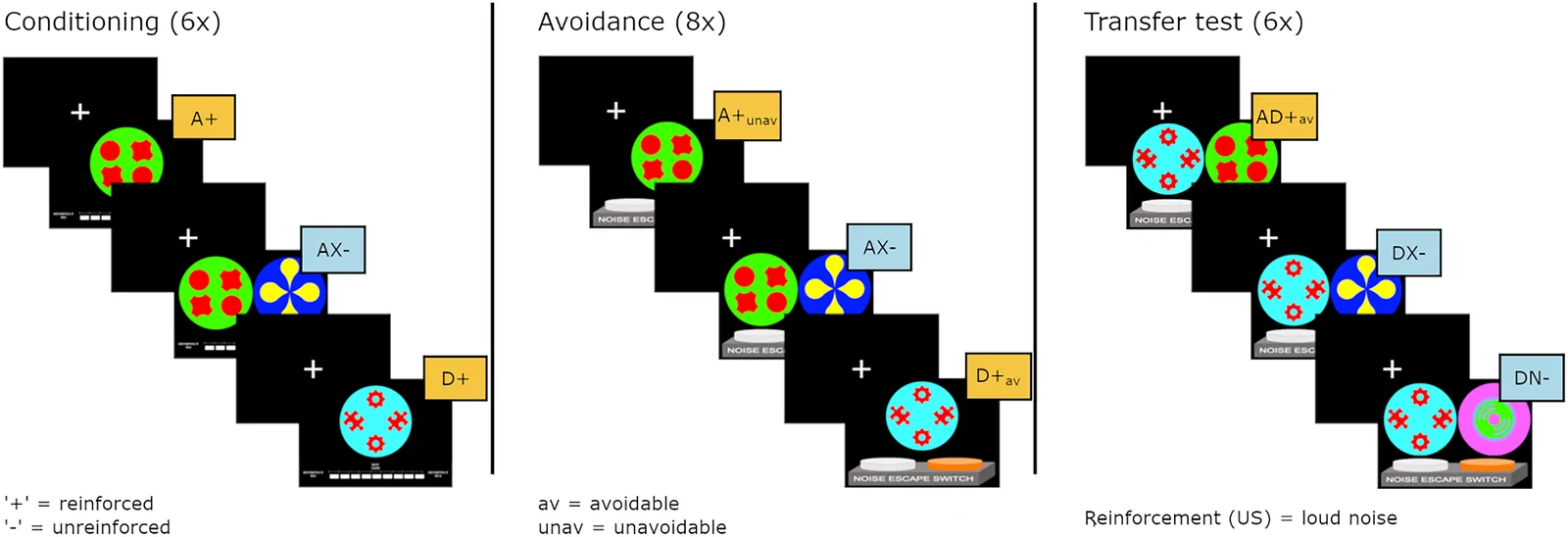
Pavlovian avoidance fMRI task. Two threat stimuli (A+ and D+) and the conditioned inhibitor (AX−) were presented during the conditioning phase, including threat expectancy ratings. During the avoidance phase, participants could avoid receiving an anticipated noise burst via button press (escape switch) for stimulus D+_av_ but not for stimulus A+_unav_. The test phase included three compound stimuli: a combination of the two threat cues (AD+_av_) and a combination of the threat cue with the conditioned inhibitor (DX−) and with a neutral stimulus (DN−) used to validate avoidance and safety learning.

Participants completed a novel avoidance learning task during fMRI (Fig. 1), adapted from our prior work (Laing et al., 2021, 2022b). The conditioned stimuli (CS) were a series of geometric figures, each presented for 5 s and the unconditioned stimulus (US) was a 95 dB white noise burst lasting for 500 ms. A white fixation cross was shown at the center of a black screen during the intertrial intervals (ITIs) with durations between 4 and 12 s (mean = 8.23 s). The task consisted of three phases: “conditioning”, “avoidance”, and “test”. Participants were briefed about the task before going into the scanner and received verbal instructions while in the scanner:

Hi [name participant], you still doing okay? Ok, the next task is the shapes task you went through earlier. You’ll see some shapes on the screen, some will be followed by a LOUD NOISE, others will be followed by NO NOISE. In the 1st phase, you’ll learn which shapes are followed by the noise by making ratings when the shapes appear using the button box. In the 2nd and 3rd phases, instead of ratings, you respond by flicking a switch that appears on screen. The switch can prevent the noise from occurring, but only for some shapes. You will have to learn when the switch prevents the noise, and when it does nothing. Just remember that you have a limited number of switch-presses, so don’t just use it on every trial, only use it when you think it will work to prevent the noise. Finally, for all trials, remember to respond quickly as you will have only 4 seconds to do so. Does that all make sense? Can you press the buttons for me to test they are working: Button 1? Button 2? Okay great. We’ll be starting soon, so remember to keep still.

During the conditioning phase, participants learned two CS+ (D+ and A+) and a compound CS− association (AX−, conditioned inhibitor). A CS+ reinforcement rate of 100% was implemented in this phase, and each CS was presented six times in a pseudorandomized order (18 total trials). Stimuli were presented alone for 1 s, followed by a threat expectancy rating scale for 4 s. Ratings were given on a 9-point scale ranging from “DEFINITELY NO” to “DEFINITELY YES” with “NOT SURE” in the middle. Participants responded with a button box in either the left or right hand (counterbalanced across participants). After the conditioning phase, participants received written instructions on the screen about the avoidance phase, which included the same three stimuli used during the conditioning phase. The instructions were the following: “In the next phase, you can press a button to cancel the noise. Your job is to press the button when you think the noise is MOST LIKELY to occur. You have a limited number of button presses available.” During this phase, participants had to learn the stimulus-avoidance associations, learning when avoidance was useful and when it was not. During avoidance, participants were presented with a noise escape switch for 4 s, allowing them to avoid the US. The noise escape switch included two buttons, a white and an orange one. At the start of each trial, a white bar would appear above the white button. To avoid the noise, participants had to “turn on” the noise escape switch by moving the white bar to the orange button using the button box. Participants were instructed that they had a limited number of button presses to induce a cost to the avoidance behavior and therefore they could also “turn off” the switch by moving the white bar back to the white button. Pressing the button was effective for one CS+ (D+_av_, avoidable stimulus), but not for the other (A+_unav_, unavoidable stimulus) and was irrelevant during AX− presentations. Each stimulus was presented eight times in pseudorandomized order (24 total trials). After the avoidance phase concluded, the screen displayed instructions stating: “Phase 2 over. In the next phase, continue to use the button when you think it is MOST LIKELY to occur.” The final “test” phase was used to validate learning outcomes from the conditioning and avoidance phases. The test phase included three different compound stimuli (AD+, DX−, DN−) with avoidance responses effective for AD+ and irrelevant for DX− and DN− presentations. The combination of avoidable with unavoidable stimuli (AD+) was used to test whether participants would avoid more or less to this stimulus. It was included as an equivalent to a transfer test to check whether the memory of D+_av_ was stronger than A+_unav_ or the other way around. If stimulus D+_av_ created a stronger memory, participants will avoid AD+ more, if A+_unav_ created a stronger memory, participants will avoid AD+ less. Both DX− and DN− served as safety stimuli, with DX− used to test the inhibitory value of X. The task was programmed in E-Prime 3.0 (Psychology Software Tools, 2016) and presented on a 32 inch LCD BOLD screen (Cambridge Research Systems), which was projected to a reverse mirror mounted to the participant’s head coil during scanning. The US noise bursts were delivered via Sensimetrics Insert Earphones (S15 model, Sensimetrics), which also provided passive noise cancellation (∼30 dB). Participants’ responses were registered using a two-button LS-PAIR Lumina response pad (Cedrus), which they were familiarized with before scanning.

### Behavioral analysis

Ratings and avoidance responses were separated into early and late trials (three trials each for conditioning and test phases and four trials each for the avoidance phase) consistent with the imaging analyses. A repeated-measures ANOVA was performed for each phase with “phase” (early, late) and “CS” as within-subjects factors. If Mauchly’s test indicated that assumptions for sphericity were violated, Greenhouse–Geisser correction was used. Effect sizes were computed using partial eta squared. Paired *t* tests were used for post hoc analyses (*p* < 0.05), with 95% confidence intervals reported and effect sizes calculated using Cohen’s *d*. Bonferroni’s correction was applied for multiple comparisons. Additionally, we used a general linear mixed model to behaviorally validate avoidance learning. The model included an interaction effect of stimulus × trial [*β*_0_ + *β*_1_ × Stimulus + *β*_2_ × Trial + β_3_ × (Stimulus × Trial)] with Stimulus A+ as the intercept and used a binomial distribution. All behavioral analyses were performed in RStudio v.2023.03.0.386 (Posit Team).

### Image acquisition

Image acquisition was performed using a 7 tesla research scanner (Siemens Healthcare) equipped with a 32-channel head coil (Nova Medical). Functional (T2*-weighted) images were obtained using a multiband- and GRAPPA-accelerated gradient-echo planar imaging (GE-EPI; Moeller et al., 2010) sequence in the steady state [multiband factor, 6; parallel reduction factor, 2; repetition time (TR), 800 ms; echo time, 22.2 ms; and flip angle, 45°; in a 20.8 cm field of view; with 130 × 130 pixel matrix; in-plane voxel size, 1.6 × 1.6 mm] with 84 interleaved slices of 1.6 mm thickness (no gap) parallel to the anterior-posterior commissure line, covering the whole brain. The total sequence time was ∼12 min for the avoidance learning task, resulting in 1,022 whole-brain EPI volumes. A high-resolution structural T1-weighted image was obtained from each participant using magnetization-prepared two rapid gradient echo sequence (MP2RAGE; Marques et al., 2010) for coregistration with the functional images (parallel reduction factor, 4; repetition time, 5 s; echo time, 3.06 ms; flip angle, 13°; in a 24 cm field of view; with a 330 × 330-pixel matrix; in-plane voxel size, 0.73 × 0.73 mm; slice thickness, 0.73 mm with 224 sagittal slices aligned parallel to the midline). To minimize head movement during scanning, foam pads were inserted on either side of the participants’ heads. Respiration and cardiac pulse were also recorded at 50 Hz during the session using a respiratory belt and pulse oximeter to be used for physiological noise correction.

### Image preprocessing

Imaging data were preprocessed using Statistical Parametric Mapping (SPM) 12 (v7771, Wellcome Trust Centre for Neuroimaging) within a MATLAB 2023a environment (The MathWorks). Motion artifacts were corrected by realigning each participant’s time series to the mean image, and all images were then resampled using fourth-degree B-spline interpolation. Individualized motion regressors were created within SPM to account for movement (Wilke, 2012). Three participants were excluded due to a mean total scan-to-scan displacement exceeding 3.2 mm (i.e., twice the size of one functional voxel). Each participant’s anatomical images were coregistered to their respective mean functional image, segmented and normalized to the International Consortium of Brain Mapping template using the unified segmentation plus Diffeomorphic Anatomical Registration Through Exponentiated Lie Algebra (DARTEL) approach. Smoothing was applied using a 3.2 mm full-width at half-maximum Gaussian kernel to preserve spatial specificity.

Physiological noise was modeled at the first level using the PhysIO Toolbox (Kasper et al., 2017). This toolbox applies noise correction to fMRI sequences using physiological recordings and has been found to improve blood oxygen level-dependent (BOLD) signal sensitivity and temporal signal-to-noise ratio at 7 tesla (Reynaud et al., 2017; see also Steward et al., 2022). The Retrospective Image-based Correction function (Glover et al., 2000) was applied to model the periodic effects of heartbeat and breathing on BOLD signals, using acquired cardiac/respiratory phase information. The respiratory response function (Birn et al., 2008), convolved with respiration volume per time, was used to model low-frequency signal fluctuations arising from changes in breathing rate and depth. Heart rate variability was convolved with a pre-defined cardiac response function (Chang et al., 2009) to account for BOLD variance due to heart rate-dependent changes in blood oxygenation. Individualized DARTEL tissue maps segmented from each participant’s respective anatomical scan were used to apply aCompCor, which modeled negative BOLD signals using six principal components derived from white matter and cerebrospinal fluid (Behzadi et al., 2007).

### fMRI task analyses

Preprocessed time series were entered into a first-level general linear model where CS onsets were convolved with the canonical hemodynamic response function and included in the model as regressors of interest. Fixation cross ITIs for the entire task were used as the implicit baseline. Time series were high-pass filtered with a cutoff period of 128 s to account for low-frequency noise. FAST was used to model temporal autocorrelations, which has been found to perform better than AR(1) for short TRs at 7 tesla (Olszowy et al., 2019).

Contrast images were estimated for each CS against the implicit baseline and used for second-level GLMs. Our first comparison of interest involved successful versus unsuccessful avoidance (D+_av_ > A+_unav_), including only A+ trials in which the participant attempted an (unsuccessful) avoidance response, thereby ensuring both conditions involved active responding. Because, overall, participants quickly learned that A+ was unavoidable, we also compared successful avoidance versus all A+ trials during the avoidance phase (D+_av_ > A+_unav all_). To examine learning changes across the avoidance phase, we compared early (first four) avoidance versus late (last four) avoidance trials (D+_av early_ > D+_av late_). Finally, we compared successful avoidance learning to safety learning, indexed by expectancy ratings with both conditions including button presses (D+_av_ > AX−_cond_). To account for potential motor confounds in the successful avoidance versus safety learning contrast, we performed a conjunction-null analysis to identify voxels showing significant activation of both D+_av_ and AX−_cond_ against the baseline. We then used the resulting map as an exclusive mask on the original D+_av_ versus AX−_cond_ contrast—retaining only voxels preferentially active during successful avoidance and not during the shared motor/sensory response. To account for potential learning confounds, we also compared late successful avoidance trials to late safety learning trials (D+_av late_ > AX−_cond_ _late_). All second-level analyses were corrected for multiple comparisons using a whole-brain false discovery rate (FDR) threshold of *p* < 0.05 with a five-voxel minimum cluster extent. We chose FDR because our analysis was discovery-oriented—aimed at characterizing distributed activation patterns associated with avoidance learning across multiple regions—for which controlling the expected proportion of false discoveries provides a more interpretable error rate than controlling the familywise probability. This approach is consistent with our previous work using comparable 7 tesla acquisitions (Laing et al., 2022b). The number of independent tests in high-resolution 7 T data is substantially larger than in conventional 3 T acquisitions due to smaller voxel sizes, which reduces statistical power under voxel-wise familywise error (FWE) control and motivates the FDR framework for whole-brain discovery analyses (Sclocco et al., 2018). Labels were identified using the SPM GLM output (AAL3 1 mm atlas and expert labelling) and were based on the cluster-level output.

## Results

### Behavioral results

**Figure 2.**
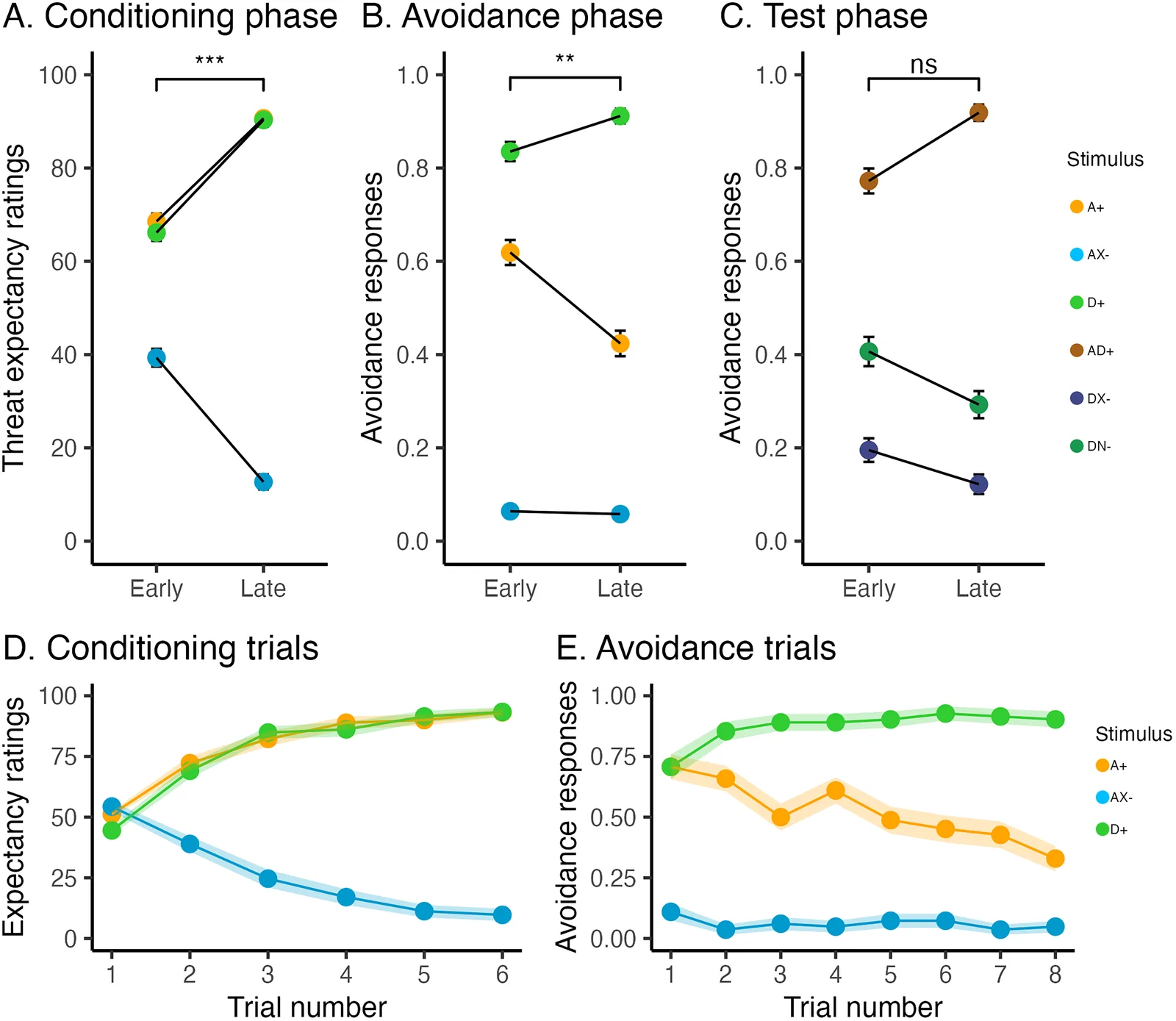
Behavioral results. ***A***, Threat expectancy ratings were higher for both threat stimuli and lower for the safety signal during late versus early conditioning. ***B***, Avoidance responses to the avoidable stimulus increased in the late versus early avoidance phase, while the unavoidable stimulus showed a decrease in responses and the safety stimulus showed no difference. ***C***, Avoidance responses showed no differences between the early and late test phase, but the responses to each stimulus were significantly different from one another. ***D***, Trial-by-trial expectancy ratings across conditioning trials. The *y*-axis illustrates average expectancy ratings. ***E***, Trial-by-trial avoidance responses across avoidance trials, indicating quick learning of successful avoidance responses. The *y*-axis shows average avoidance responses. A+ was unavoidable and D+ was avoidable. Extended Data Figure 2-1 supports this figure.

Threat expectancy ratings during the conditioning phase showed a main effect of stimulus (*F*_(2,162)_ = 269.77, *p* < 0.001, *η*_p_^2^ = 0.77); a main effect of phase (*F*_(1,81)_ = 45.72, *p* < 0.001, *η*_p_^2 ^= 0.36); and a stimulus × phase interaction (*F*_(2,162)_ = 169.23, *p* < 0.001, *η*_p_^2 ^= 0.68). Post hoc tests indicated significantly higher threat expectancies for both stimulus A+ (mean = 53.6, *t* = 17.41, *d* = 1.07, *p* < 0.001, 95% CI [46.08, 61.13]) and stimulus D+ (mean = 52.23, *t* = 17.39, *d* = 1.02, *p* < 0.001, 95% CI [44.89, 59.57]) compared with the conditioned inhibitor AX−. Figure 2*A* shows higher expectancy ratings in the late phase compared with the early phase for stimulus D+ (mean = 24.15, *t* = 12.17, *d* = 0.77, *p* < 0.001, 95% CI [18.15, 30.15]) and stimulus A+ (mean = 22.12, *t* = 12.55, *d* = 0.81, *p* < 0.001, 95% CI [16.79, 27.45]), while expectancy ratings were lower for stimulus AX− (mean = −26.64, *t* = −11.32, *d* = −0.79, *p* < 0.001, 95% CI [−33.76, −19.52]). These findings indicate successful acquisition of fear and safety learning.

10.1523/ENEURO.0170-26.2026.f2-1 Figure 2-1 Discriminative avoidance learning. The plot illustrates average discriminative avoidance learning for D + _av_ and A + _unav_, indicating an increase in avoidance responses for D + _av_ and decrease for A + _uanv_. The y-axis illustrates the difference in avoidance responses for D + _av_ minus A + _unav_, with -1 corresponding to avoiding A + _unav_ but not D + _av_, 0 to no avoidance for either stimulus, and 1 to avoiding D + _av_ but not A + _unav_. Download Figure 2-1, TIF file.

Avoidance responses during the avoidance phase also showed a main effect of stimulus (*F*_(2,162)_ = 239.7, *p* < 0.001, *η*_p_^2^ = 0.75); a main effect of phase (*F*_(1,81)_ = 11.65, *p* = 0.001, *η*_p_^2 ^= 0.13); and a stimulus × phase interaction (*F*_(2,162)_ = 31.68, *p* < 0.001, *η*_p_^2 ^= 0.28). Figure 2*B* shows a significant decrease in responding to stimulus A+ in the late phase compared with the early phase (mean = −0.20, *t* = −6.56, *d* = 0.33, *p* < 0.001, 95% CI [−0.29, −0.11]), while responses to stimulus D+ increased from the early to the late phase (mean = 0.08, *t* = 3.07, *d* = −0.17, *p* = 0.04, 95% CI [0, 0.15]). These results confirm that participants successfully acquired the appropriate avoidance responses.

To behaviorally validate avoidance learning, we used a general linear mixed model. The results indicated that, while initially there was no significant difference in avoidance responses between stimulus A+ and stimulus D+ (*β* = 0.13, *z* = 0.42, *p* = 0.67), this difference became significant in the following trials as evidenced by an interaction effect of stimulus D × Trial (*β* = 0.43, *z* = 6.4, *p* < 0.001). Figure 2*E* illustrates this divergence, with responses to D+ increasing as responses to A+ decrease. This was also reflected when examining discriminative avoidance learning (D+ minus A+; Extended Data Fig. 2-1).

The test phase analyses indicated a main effect of stimulus (*F*_(2,162)_ = 124.84, *p* < 0.001, *η*_p_^2 ^= 0.61), but not phase (*F*_(1,81)_ = 0.64, *p* = 0.43), and an interaction effect of stimulus × phase (*F*_(2,162)_ = 29.61, *p* < 0.001, *η*_p_^2 ^= 0.27). Post hoc tests indicated higher responses to AD+ compared with both DX− (mean = 0.69, *t* = 18, *d* = 1.28, *p* < 0.001, 95% CI [0.60, 0.78]) and DN− (mean = 0.50, *t* = 10.76, *d* = 0.39, *p* < 0.001, 95% CI [0.38, 0.61]). In addition, Figure 2*C* shows that responses to DN− were significantly higher than to DX− (mean = 0.19, *t* = 3.85, *d* = 0.36, *p* < 0.001, 95% CI [0.07, 0.31]). These findings validate participants’ avoidance and safety learning.

### Imaging results

#### Successful versus unsuccessful avoidance

**Figure 3.**
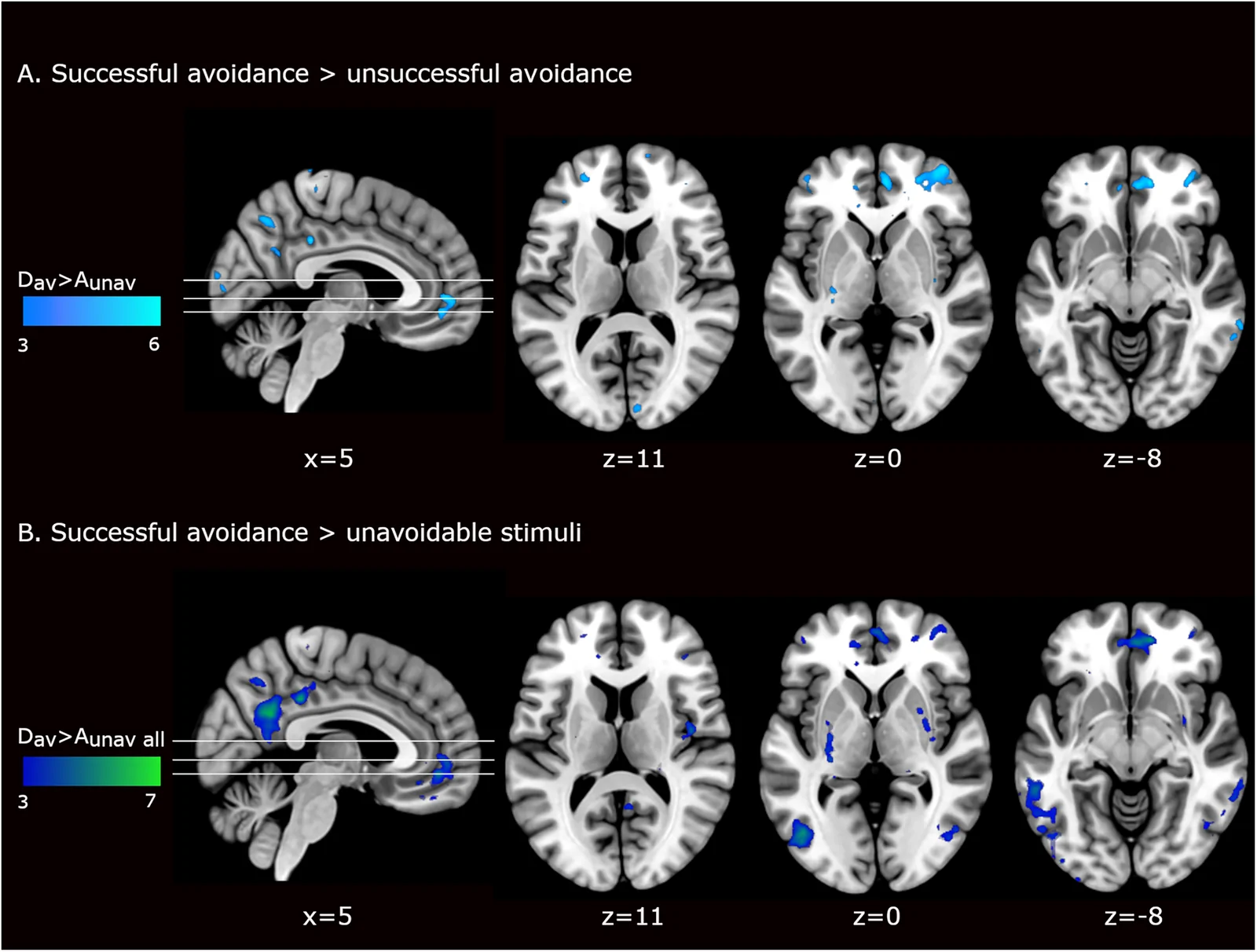
Significant whole-brain fMRI results of successful versus unsuccessful avoidance. Panel ***A*** shows brain regions with significantly greater activation during successful versus unsuccessful avoidance (D+_av_ > A+_unav_). Whole-brain FDR-corrected (*p* < 0.05) results are displayed on a template in MNI space. Panel ***B*** demonstrates brain regions with significantly greater activation during successful avoidance versus all unavoidable stimuli (D+_av_ > A+_unav all_). Extended Data Figures 3-1 and 3-2 support this figure.

As shown in Figure 3*A*, successful avoidance was associated with significantly greater activation of the vmPFC (*x*, *y*, *z* = 13, 46, −8; *T* = 5.44; *p*_FDR_ = 0.008; 237 voxels) spanning the ventral anterior cingulate cortex (ACC), the ventral posterior cingulate (PCC; *x*, *y*, *z* = 5, −34, 35; *T* = 5.47; *p*_FDR_ = 0.008; 20 voxels), the precuneus (*x*, *y*, *z* = 5, −62, 48; *T* = 4.85; *p*_FDR_ = 0.011; 78 voxels), and the ventrolateral prefrontal cortex (*x*, *y*, *z* = 37, 53, −3; *T* = 5.53; *p*_FDR_ = 0.008; 578 voxels). Additional regions included the right primary motor cortex, bilateral middle temporal gyrus, right cuneus, and bilateral inferior parietal gyrus, among others. Complete results are provided in Extended Data Figure 3-1. To supplement these findings, we compared successful avoidance to all unavoidable trials (D+_av_ > A+_unav all_). Results were broadly replicative in nature (Fig. 3*B*), with additional involvement of the bilateral insular cortex, left caudate, right putamen, and middle occipital gyrus. Complete results are provided in Extended Data Figure 3-2.

10.1523/ENEURO.0170-26.2026.f3-1 Figure 3-1 *Differential responses to successful (D* *+* *_av_) versus unsuccessful avoidance (A* *+* *_unav_)* ^a^*K_E_*, cluster size in number of voxels; *T*, SPM T-scores; IFG, inferior frontal gyrus; L, left; R, right. Coordinates reported in MNI space. Whole-brain contrasts estimated at *P*_FDR_<0.05. Download Figure 3-1, DOCX file.

10.1523/ENEURO.0170-26.2026.f3-2 Figure 3-2 *Differential responses to successful avoidance (D* *+* *_av_) versus unavoidable stimuli (A* *+* *_unav all_)* ^a^*K_E_*, cluster size in number of voxels; *T*, SPM T-scores; L, left; R, right. Coordinates reported in MNI space. Whole-brain contrasts estimated at *P*_FDR_<0.05. Download Figure 3-2, DOCX file.

#### Early versus late avoidance learning

**Figure 4.**
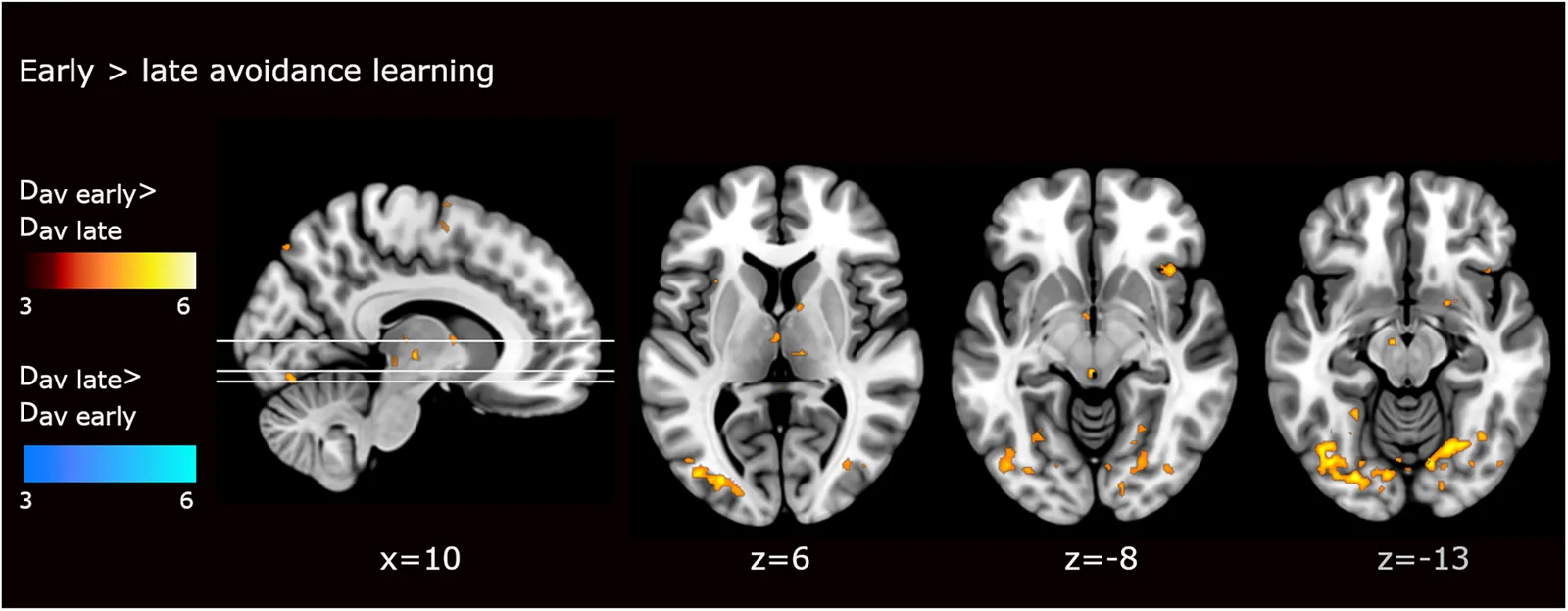
Significant whole-brain fMRI results of early versus late avoidance learning. Brain regions with significantly greater activation during early versus late avoidance trials (D+_av early_ > D+_av late_). Whole-brain FDR-corrected (*p* < 0.05) results are displayed on a template in MNI space. Extended Data Figure 4-1 supports this figure.

As shown in Figure 4, early versus late trials of successful avoidance (D+_av early_ > D+_av late_) evoked significantly greater activation of the right ventral lateral, right mediodorsal and right lateral pulvinar thalamic nuclei; midbrain periaqueductal gray (PAG) and left substantia nigra pars compacta (SNc), bilateral insula, right caudate nucleus, and the visual association cortex spanning the middle and inferior occipital, fusiform, and lingual gyrus. Activation in the thalamus was represented by three significant clusters: one located more ventrally (*x*, *y*, *z* = 13, −16, −2; *T* = 4.87; *p*_FDR_ = 0.006; 28 voxels), one located more dorsally (*x*, *y*, *z* = 0, −13, 3; *T* = 4.27; *p*_FDR_ = 0.013; 21 voxels), and one located more laterally (*x*, *y*, *z* = 11, −21, 6; *T* = 4.4; *p*_FDR_ = 0.011; 27 voxels). Activation in the PAG was represented by one significant cluster (*x*, *y*, *z* = 0, −29, −8; *T* = 4.69; *p*_FDR_ = 0.008; 21 voxels), and activation in the SNc was represented by one significant cluster (*x*, *y*, *z* = −6, −14, −13; *T* = 4.55; *p*_FDR_ = 0.009; 8 voxels).

10.1523/ENEURO.0170-26.2026.f4-1 Figure 4-1 *Avoidance learning: differential responses to successful early (D* *+* *_av early_) versus late avoidance (D* *+* *_av late_) learning and late versus early avoidance learning* ^a^*K_E_*, cluster size in number of voxels; *T*, SPM T-scores; L, left; R, right. Coordinates reported in MNI space. Whole-brain contrasts estimated at *P*_FDR_<0.05. Download Figure 4-1, DOCX file.

Comparing late avoidance to early avoidance trials (D+_av late_ > D+_av early_), we observed significantly greater activation of the left primary motor cortex, right cerebellum (VI), left dorsolateral prefrontal cortex (dlPFC), right inferior parietal gyrus, and left paracentral lobule. Complete results are provided in Extended Data Figure 4-1.

#### Avoidance versus safety learning

**Figure 5.**
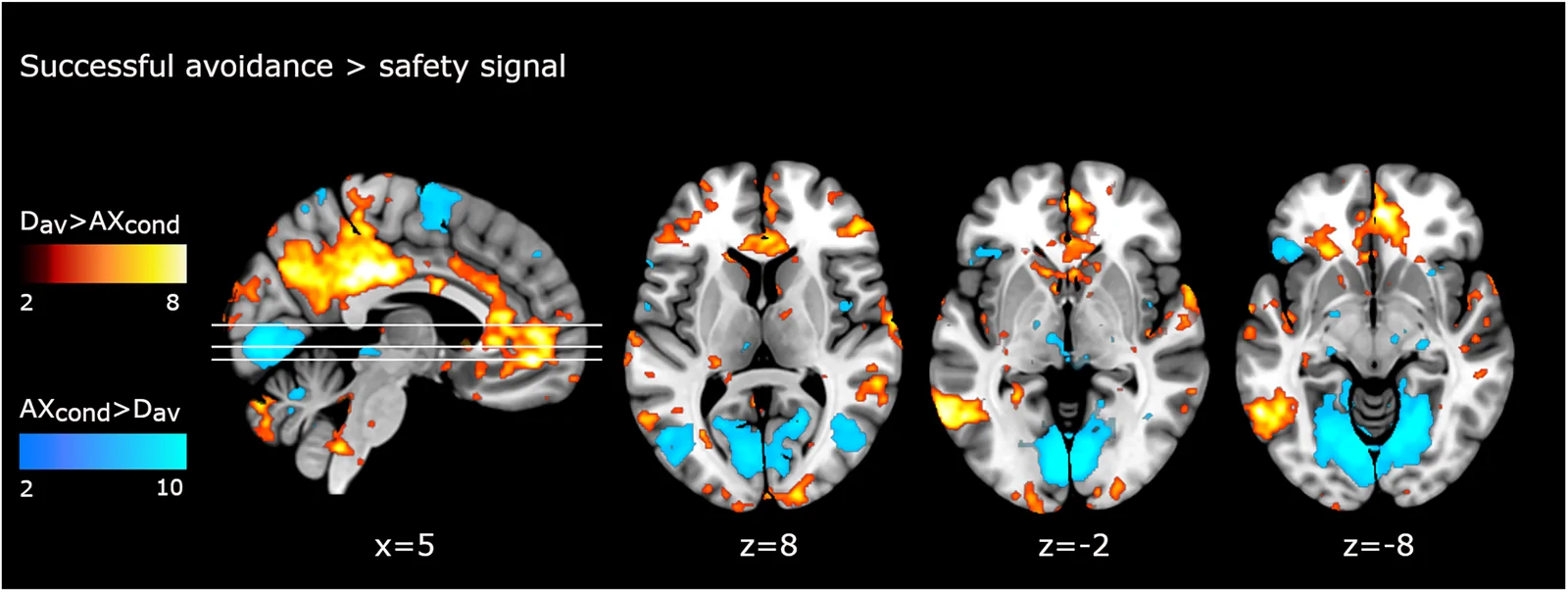
Significant whole-brain fMRI results of successful avoidance versus safety learning. Brain regions with significantly greater activation during successful avoidance versus safety trials (D+_av_ > AX−_cond_) and to safety versus successful avoidance trials (AX−_cond_ > D+_av_). Whole-brain FDR-corrected (*p* < 0.05) results are displayed on a template in MNI space. Extended Data Figures 5-1–5-6 support this figure.

Figure 5 shows the comparison between successful avoidance and the safety signal (D+_av_ > AX−_cond_), which identified greater activation across several regions: the right vmPFC, extending into the ACC; the PCC, extending into the precuneus; the somatosensory cortex and primary motor cortex, the dlPFC, the right nucleus accumbens (NAc), bilateral caudate, the ventrolateral thalamic nucleus, the middle temporal gyrus, and the right medial and posterior orbitofrontal cortex (OFC). Activation in the vmPFC was represented by one significant cluster (*x*, *y*, *z* = 3, 45, −8; *T* = 6.87; *p*_FDR_ < 0.001; 4,919 voxels), and activation in the PCC was represented by one significant cluster located more ventrally (*x*, *y*, *z* = 5, −30, 34; *T* = 7.48; *p*_FDR_ < 0.001; 9,807 voxels). Activation in the NAc was represented by one significant cluster (*x*, *y*, *z* = 6, 6, −10; *T* = 3.73; *p*_FDR_ = 0.008; 15 voxels), and activation in the OFC was represented by two significant clusters: one located more posterior bordering the insula (*x*, *y*, *z* = 32, 30, −16; *T* = 3.33; *p*_FDR_ = 0.016; 9 voxels) and one cluster located more medial (*x*, *y*, *z* = 18, 29, −21; *T* = 3.38; *p*_FDR_ = 0.015; 7 voxels). Complete results are provided in Extended Data Figure 5-1. To account for motor responses, we performed the same contrast analysis masked with the results of a conjunction-null analysis of D+_av_ and AX−_cond_ and found that the results were very similar to the original contrast (Extended Data Figs. 5-2, 5-3). To account for potential learning confounds, we also analyzed late successful avoidance versus late safety learning trials. The results of this contrast broadly paralleled the results of the original contrast (Extended Data Figs. 5-4, 5-5).

10.1523/ENEURO.0170-26.2026.f5-1 Figure 5-1 *Differential responses to successful avoidance (D* *+* *_av_) versus safety learning (AX-_cond_)* ^a^*K_E_*, cluster size in number of voxels; *T*, SPM T-scores; L, left; R, right. Coordinates reported in MNI space. Whole-brain contrasts estimated at *P*_FDR_<0.05. Download Figure 5-1, DOCX file.

10.1523/ENEURO.0170-26.2026.f5-2 Figure 5-2 Significant whole-brain fMRI results of successful avoidance versus safety learning with conjunction mask. Brain regions with significantly greater activation during successful avoidance trials versus safety learning trials (D + _av_ > AX-_cond_) masked with the results of a conjunction-null analysis of D + _av_ and AX-_cond_. Whole-brain FDR-corrected (*P* < 0.05) results are displayed on a template in MNI space. Download Figure 5-2, TIF file.

10.1523/ENEURO.0170-26.2026.f5-3 Figure 5-3 *Differential responses to successful avoidance (D* *+* *_av_) versus safety learning (AX-_cond_) masked by the results of a conjunction-null analysis of D* *+* *_av_ and AX-_cond_* ^a^*K_E_*, cluster size in number of voxels; *T*, SPM T-scores; L, left; R, right. Coordinates reported in MNI space. Whole-brain contrasts estimated at *P*_FDR_<0.05. Download Figure 5-3, DOCX file.

10.1523/ENEURO.0170-26.2026.f5-4 Figure 5-4 Significant whole-brain fMRI results of late successful avoidance versus late safety learning. Brain regions with significantly greater activation during late successful avoidance trials versus late safety learning trials (D + _av late_ > AX-_cond late_). Whole-brain FDR-corrected (*P* < 0.05) results are displayed on a template in MNI space. Download Figure 5-4, TIF file.

10.1523/ENEURO.0170-26.2026.f5-5 Figure 5-5 *Differential responses to late successful avoidance trials (D* *+* *_av late_) versus late safety learning trials (AX-_cond late_)* ^a^*K_E_*, cluster size in number of voxels; *T*, SPM T-scores; IFG, inferior frontal gyrus; L, left; R, right. Coordinates reported in MNI space. Whole-brain contrasts estimated at *P*_FDR_<0.05. Download Figure 5-5, DOCX file.

Safety versus successful avoidance (AX−_cond_ > D+_av_) identified greater activation of the visual association cortex, spanning the lingual and fusiform gyrus, and the calcarine fissure; the medial temporal gyrus, the medial pulvinar, ventral lateral, lateral geniculate, and intralaminar thalamic nucleus; the right insula, the OFC, the left hippocampus, and the right putamen. Complete results are provided in Extended Data Figure 5-6.

10.1523/ENEURO.0170-26.2026.f5-6 Figure 5-6 *Differential responses to safety learning (AX-_cond_) versus successful avoidance (D* *+* *_av_)* ^a^*K_E_*, cluster size in number of voxels; *T*, SPM T-scores; IFG, inferior frontal gyrus; L, left; R, right. Coordinates reported in MNI space. Whole-brain contrasts estimated at *P*_FDR_<0.05. Download Figure 5-6, DOCX file.

## Discussion

We aimed to characterize the neural circuitry of avoidance learning using a novel avoidance learning paradigm combined with 7 tesla fMRI. Behaviorally, participants demonstrated rapid avoidance learning, indicated by discriminating between threat cues associated with successful avoidance versus unavoidable threat delivery. Consistent with our predictions, early avoidance learning distinctly engaged midbrain-striatal and insular regions—a pattern indicative of active learning-related processing during the initial formation of avoidance associations. However, we did not find the hypothesized vmPFC activity in the late avoidance trials. Established avoidance responses engaged cortical “safety-responsive” brain regions, including the vmPFC and PCC—an effect that persisted even when established avoidance was directly compared with pavlovian safety learning. These findings indicate that established avoidance relies on cortical safety-responsive regions, including the vmPFC, whereas initial avoidance recruited additional midbrain-striatal regions and threat-responsive circuitry compared with late avoidance. This pattern parallels recent evidence from safety learning studies (Laing et al., 2021, 2022b), suggesting a common neural substrate underlying both processes.

Behaviorally, participants showed both successful acquisition of pavlovian safety learning—associating certain cues with threat omission (AX−)—and active avoidance learning, where the avoidance response became associated with threat omission (successful avoidance, D+_av_). The latter were noted to occur rapidly, typically within the first avoidance learning trials. Critically, avoidance responses remained appropriately selective. Responses increased and were maintained for one stimulus (D+_av_, successful avoidance), for which avoidance successfully prevented threat, and diminished for the stimulus signaling inevitable threat (A+_unav_, unsuccessful avoidance), regardless of avoidance responses. At test, this selectivity remained elevated—with appropriate avoidance responses to the threat stimulus (AD+_av_)—and did not inappropriately generalize to safety signals (DX− and DN−). Previous studies have reported a complex relationship between avoidance and safety learning: for instance, when avoidance responses are available during fear extinction, they can impair extinction success (“the protection from extinction effect”; Lovibond et al., 2009) and avoidance responses show robust renewal after extinction when the avoidance option is reintroduced (Vervliet and Indekeu, 2015). Our results instead indicate that when avoidance is learned and expressed after safety associations are formed—rather than during extinction of established fear associations—avoidance responses can remain appropriately constrained and do not interfere with safety discrimination. This suggests that the relationship between avoidance and safety learning likely depends critically on the learning context.

Established avoidance responses (when compared with unsuccessful avoidance) engaged cortical regions consistently implicated in safety learning, including the vmPFC and PCC. These regions are reliably activated by learned safety signals across differential conditioning, reversal learning, fear extinction, and extinction recall paradigms (Fullana et al., 2016; Harrison et al., 2017; Gerlicher et al., 2018; Savage et al., 2020; Esser et al., 2021). Additionally, the vmPFC has been linked to predicting a change in avoidance behavior, with more vmPFC activation correlating with a greater increase in avoidance behavior to avoidable stimuli versus unavoidable stimuli (Wang and Delgado, 2021). The vmPFC is particularly linked to positive affective processing and the representation of safety value (Zhang et al., 2015; Harrison et al., 2017), consistent with the relief and reward-like properties associated with successful avoidance. When directly comparing successful avoidance to safety learning, we observed additional engagement of reward-related regions including the ventral striatum (NAc) and medial OFC. These regions have been implicated in previous avoidance studies (Kim et al., 2006; Levita et al., 2012; Oleson et al., 2012) and align with behavioral evidence that avoiding an aversive outcome induces feelings of pleasant relief that reinforce avoidance behaviors (Vervliet et al., 2017; Leng et al., 2024). This pattern suggests that while both safety learning and active avoidance engage safety-processing circuits, successful avoidance may confer additional reward-like properties—a distinction that could contribute to the persistent nature of avoidance behaviors in anxiety disorders.

Early avoidance trials specifically engaged midbrain-striatal regions including the SNc, along with threat-responsive areas such as the PAG, mediodorsal thalamus, and anterior insula. This activation pattern during initial learning is consistent with prediction error-like processing, wherein threat expectations are reconciled with the unexpected safety outcomes produced by successful avoidance responses (Gerlicher et al., 2018; Kalisch et al., 2019; Esser et al., 2021; Laing et al., 2022b). The SNc provides dopaminergic input to the striatum (Lanciego et al., 2012) and has been implicated in signaling prediction errors—mismatches between expected and actual outcomes (Schultz, 2016). Specifically, SNc dopamine neurons show elevated firing during initial learning of unexpected positive outcomes, with activity decreasing as outcomes become fully predicted (Hollerman and Schultz, 1998)—consistent with a teaching signal. Importantly, animal studies have demonstrated that blocking dopamine release in the SNc impairs avoidance learning (Dombrowski et al., 2013; Stelly et al., 2019), and recent evidence suggests this dopamine signal may represent a “safety prediction error” that is anatomically distinct from general reward prediction errors (Duvarci, 2024; Laing et al., 2024).

The engagement of threat-responsive regions (PAG, insula, mediodorsal thalamus, dorsal striatum) during early avoidance learning likely reflects the threat associations acquired during the conditioning phase. This is consistent with other work indicating that avoidance is a dynamic process involving a shift from freezing—the physiological response during fear conditioning—to action preparation (Löw et al., 2015). The PAG is critically involved in defensive behaviors, especially when threat is imminent (Mobbs et al., 2007; Wendt et al., 2017), and receives input from threat-detection circuits (Lefler et al., 2020), while the insula and thalamus are consistently implicated in threat appraisal and the formation of stimulus-threat associations (Fullana et al., 2016; Badarnee et al., 2025a,b). Connections between the PAG and medial PFC (mPFC) have also been specifically implicated in the regulation of defensive responses (Qi et al., 2018; Lefler et al., 2020), with a shift to mPFC activity when the defensive action is cognitively processed. The involvement of the dorsal striatum is consistent with other avoidance studies (Delgado et al., 2009; Collins et al., 2014; Boeke et al., 2017), where the coupling between the striatum and mPFC has also been implicated in predicting avoidance behavior (Collins et al., 2014). Together, these findings suggest that initial avoidance learning relies on subcortical regions that process previously acquired threat associations and relay this information to cortical regions, which integrate threat and safety inputs to guide avoidance responses.

Several methodological considerations should be noted. First, while trial numbers were balanced for the successful-versus-unsuccessful and early-versus-late avoidance contrasts, the comparison of successful avoidance with pavlovian safety (D+_av_ vs AX−_cond_) differed in motor demand. To assess whether shared sensorimotor processes contributed to this contrast, we performed a conjunction-null analysis and used the result as an exclusive mask on the original contrast; the regions central to our interpretation (vmPFC, PCC, NAc, OFC) survived this masking, indicating the effects are not driven by motor demand. Second, the unavoidable stimulus (A+) appeared in compound with the safety cue (AX−) during conditioning while D+ did not, introducing an asymmetry in conditional safety probability between the two threat cues. However, end-of-conditioning threat expectancy ratings were comparable for A+ and D+, suggesting this asymmetry did not substantially shape participants’ threat appraisals. Third, we adopted whole-brain FDR correction rather than voxel-wise FWE correction. These approaches control different error rates: FDR limits the expected proportion of false positives among significant voxels, while FWE limits the probability of any false positive across the whole brain. FDR is therefore more appropriate for discovery-oriented whole-brain analyses but yields a higher absolute number of false positives than FWE at the same nominal alpha. Smaller clusters and effects close to threshold should therefore be interpreted with appropriate caution pending independent replication. Fourth, we did not include brain–behavior correlations or independent converging measures to constrain the functional interpretation of our findings. Our hypotheses were grounded in the existing pavlovian safety-learning acquisition-expression framework (Laing et al., 2022a), but we acknowledge that the mechanistic conclusions remain provisional pending future work incorporating trial-by-trial affective ratings, psychophysiological measures, or computational individual-difference modeling. Finally, we did not include a delayed test session to examine consolidation. Given that dopaminergic circuits also contribute to safety memory consolidation (Gerlicher et al., 2018; Kalisch et al., 2019), future studies should examine whether midbrain-striatal engagement during early avoidance learning predicts the persistence of avoidance over long-term (i.e., 24 h, weeks, months) training intervals.

In summary, our findings reveal a temporal dissociation in the neural circuitry of human avoidance learning: initial avoidance learning preferentially engages subcortical threat-responsive and midbrain-striatal regions, while established avoidance responses engage cortical safety-processing systems, including the vmPFC and PCC. This dissociation broadly parallels our recent findings in safety learning via conditioned inhibition, suggesting common neural substrates of avoidance and safety learning. Critically, the engagement of hypothesized learning-related midbrain-striatal circuits during early avoidance learning identifies a potential mechanistic window during which avoidance associations may be most amenable to intervention, thus preventing the consolidation of maladaptive avoidance patterns. Future research examining whether altered engagement of these early learning circuits predicts treatment resistance or relapse may inform the development of mechanistically targeted interventions, including preventative strategies.

## Synthesis

Reviewing Editor: James Howard, Brandeis University Decisions are customarily a result of the Reviewing Editor and the peer reviewers coming together and discussing their recommendations until a consensus is reached. When revisions are invited, a fact-based synthesis statement explaining their decision and outlining what is needed to prepare a revision will be listed below. The following reviewer(s) agreed to reveal their identity: NONE. Note: If this manuscript was transferred from JNeurosci and a decision was made to accept the manuscript without peer review, a synthesis may not be available.

The authors have addressed the reviewers' concerns. In explaining the use of FDR correction, however, there appears to be a misunderstanding. The reviewers don't see a principled problem with FDR correction when properly described - they are only concerned about the terminology in the stated motivation. This is a minor point and requires only small textual changes.

The authors appear to argue that FWE correction has low statistical power, in particular for 7 T data. In saying this, they appear to conflate the concepts of "conservative" with "statistical power", but the two are not necessarily related (i.e. a less "conservative" test does not necessarily have higher statistical power). The term "conservative" refers to the false positive rate (i.e. maximum rejection probability under the null hypothesis), whereas statistical power refers to the false negative rate (i.e. rejection probability under a specific distribution within the alternative hypothesis). The term "overly conservative" makes little (statistical) sense. An alpha test is simply a test for which the false positive rate never exceeds the chosen alpha level under the null hypothesis. An alpha test is said to be conservative if this is the case even if the true data distribution deviates from the parametric assumptions of the test. One cannot be "too conservative" here.

However, there is of course a problem with low statistical power, especially in a discovery setting.

For example, the problem with Bonferroni correction that the authors allude to is that there exists a uniformly more powerful alternative with the same alpha level, that is, a test that is as "conservative" but has higher statistical power for any distribution under the alternative hypothesis (this is the Holm-Bonferroni correction).

Using FDR, the authors choose a different testing framework, i.e. they don't limit the false positive rate, in order to boost statistical power.

On a different note, it would be interesting if the authors could expand a little bit why they think statistical power of FWE correction is a bigger problem for 7T than for 3T data.

## Author Response

Synthesis of Reviews:

Computational Neuroscience Model Code Accessibility Comments for Author (Required):

N/A Synthesis Statement for Author (Required):

The authors have addressed the reviewers' concerns. In explaining the use of FDR correction, however, there appears to be a misunderstanding. The reviewers don't see a principled problem with FDR correction when properly described - they are only concerned about the terminology in the stated motivation. This is a minor point and requires only small textual changes.

The authors appear to argue that FWE correction has low statistical power, in particular for 7 T data. In saying this, they appear to conflate the concepts of "conservative" with "statistical power", but the two are not necessarily related (i.e. a less "conservative" test does not necessarily have higher statistical power). The term "conservative" refers to the false positive rate (i.e. maximum rejection probability under the null hypothesis), whereas statistical power refers to the false negative rate (i.e. rejection probability under a specific distribution within the alternative hypothesis). The term "overly conservative" makes little (statistical) sense. An alpha test is simply a test for which the false positive rate never exceeds the chosen alpha level under the null hypothesis. An alpha test is said to be conservative if this is the case even if the true data distribution deviates from the parametric assumptions of the test. One cannot be "too conservative" here.

However, there is of course a problem with low statistical power, especially in a discovery setting.

For example, the problem with Bonferroni correction that the authors allude to is that there exists a uniformly more powerful alternative with the same alpha level, that is, a test that is as "conservative" but has higher statistical power for any distribution under the alternative hypothesis (this is the Holm-Bonferroni correction).

Using FDR, the authors choose a different testing framework, i.e. they don't limit the false positive rate, in order to boost statistical power.

On a different note, it would be interesting if the authors could expand a little bit why they think statistical power of FWE correction is a bigger problem for 7T than for 3T data.

Response: We thank the Editor for the helpful clarification regarding our statistical reasoning. We agree that our previous wording conflated 'conservativeness' (control of false positives under the null) with 'statistical power' (rejection probability under the alternative), and that FDR and FWE represent different testing frameworks controlling different error rates rather than more- and less-powerful versions of the same procedure.

We have revised the methods (p. 10) to frame the choice of FDR explicitly as a decision about which error rate is most appropriate to control given our discovery-oriented analysis goals, rather than as a criticism of FWE. We have also removed references to FWE being 'overly conservative.' In addition, we have added a brief explanation of why FWE power is a greater concern at 7T than at 3T - namely, that the substantially smaller voxel sizes used in 7T acquisitions increase the number of independent tests across the brain, which reduces power under voxel-wise FWE control. The limitations section (p. 18) has been updated accordingly to characterise FDR and FWE in terms of the distinct error rates they control. "...All second-level analyses were corrected for multiple comparisons using a whole-brain false discovery rate (FDR) threshold of P<0.05 with a 5-voxel minimum cluster extent. We chose FDR because our analysis was discovery-oriented - aimed at characterising distributed activation patterns associated with avoidance learning across multiple regions - for which controlling the expected proportion of false discoveries provides a more interpretable error rate than controlling the family-wise probability. This approach is consistent with our previous work using comparable 7-Tesla acquisitions (Laing et al., 2022b). The number of independent tests in high-resolution 7T data is substantially larger than in conventional 3T acquisitions due to smaller voxel sizes, which reduces statistical power under voxel-wise family-wise error (FWE) control and motivates the FDR framework for whole-brain discovery analyses (Sclocco et al., 2018)..." "...Third, we adopted whole-brain FDR correction rather than voxel-wise FWE correction. These approaches control different error rates: FDR limits the expected proportion of false positives among significant voxels, while FWE limits the probability of any false positive across the whole brain. FDR is therefore more appropriate for discovery-oriented whole-brain analyses but yields a higher absolute number of false positives than FWE at the same nominal alpha. Smaller clusters and effects close to threshold should therefore be interpreted with appropriate caution pending independent replication..."
