# Supplementary material for: The Neural Basis of Avoidance Learning and Its Relation to Safety Processing
Source: eNeuro. 2026 Aug 25;13(8):ENEURO.0170-26.2026. doi: 10.1523/ENEURO.0170-26.2026 (PMC13528696; doi:10.1523/ENEURO.0170-26.2026)
Supplement: Supplementary file 1 [file eneuro-13-ENEURO.0170-26.2026-s002.docx]

**Figure 3-1**

*Differential responses to successful (D+_av_) versus unsuccessful avoidance (A+_unav_)*

|  | MNI coordinates | | |  |  |
| --- | --- | --- | --- | --- | --- |
| Regions | x | y | z | *K_E_* | *T* |
| Successful > unsuccessful avoidance |  |  |  |  |  |
| Postcentral gyrus, R | 61 | -5 | 32 | 211 | 5.60 |
| Middle temporal gyrus, L | -59 | -40 | -11 | 82 | 5.59 |
| Middle frontal gyrus, R | 37 | 53 | -3 | 578 | 5.53 |
| Middle cingulate & paracingulate gyri, R | 5 | -34 | 35 | 20 | 5.47 |
| Superior frontal gyrus, medial orbital, R | 13 | 46 | -8 | 237 | 5.44 |
| Postcentral gyrus, L | -51 | -11 | 27 | 186 | 5.34 |
| Cuneus, R | 10 | -90 | 14 | 127 | 5.24 |
| Inferior parietal gyrus, L | -26 | -59 | 37 | 57 | 5.18 |
| Superior frontal gyrus, dorsolateral, R | 29 | 26 | 53 | 340 | 5.00 |
| Inferior temporal gyrus, R | 58 | -27 | -22 | 25 | 4.99 |
| Angular gyrus, R | 51 | -62 | 29 | 222 | 4.96 |
| Middle frontal gyrus, L | -30 | 45 | 8 | 86 | 4.95 |
| Superior frontal gyrus, dorsolateral, L | -24 | 50 | 10 | 47 | 4.93 |
| Middle frontal gyrus, L | -40 | 19 | 54 | 123 | 4.92 |
| Postcentral gyrus, R | 37 | -32 | 48 | 25 | 4.86 |
| Precuneus, R | 5 | -62 | 48 | 78 | 4.85 |
| Middle temporal gyrus, R | 67 | -38 | -8 | 23 | 4.84 |
| Inferior parietal gyrus, R | 37 | -48 | 54 | 34 | 4.83 |
| Crus II of cerebellar hemisphere, L | -42 | -66 | -40 | 98 | 4.77 |
| Inferior temporal gyrus, R | 51 | -14 | -27 | 27 | 4.73 |
| Lobule III of cerebellar hemisphere, R | 19 | -27 | -24 | 12 | 4.72 |
| Superior frontal gyrus, dorsolateral, R | 16 | 43 | 46 | 17 | 4.64 |
| Precuneus, R | 8 | -54 | 27 | 80 | 4.56 |
| Postcentral gyrus, R | 46 | -35 | 62 | 50 | 4.56 |
| Crus I of cerebellar hemisphere, R | 40 | -62 | -40 | 19 | 4.52 |
| Superior frontal gyrus, medial, R | 14 | 62 | 13 | 12 | 4.49 |
| Middle frontal gyrus, L | -22 | 37 | -11 | 8 | 4.44 |
| Lingual gyrus, L | -14 | -99 | -14 | 57 | 4.43 |
| Middle frontal gyrus, R | 48 | 30 | 34 | 24 | 4.43 |
| Middle temporal gyrus, R | 64 | -46 | -8 | 11 | 4.38 |
| Middle temporal gyrus, R | 64 | 2 | -19 | 9 | 4.35 |
| Angular gyrus, L | -40 | -70 | 37 | 20 | 4.34 |
| Middle temporal gyrus, L | -61 | -14 | -24 | 9 | 4.32 |
| Inferior temporal gyrus, R | 61 | -35 | -18 | 20 | 4.28 |
| Paracentral lobule, R | 2 | -30 | 69 | 36 | 4.26 |
| Superior frontal gyrus, medial orbital, L | -6 | 43 | -8 | 10 | 4.23 |
| Inferior temporal gyrus, L | -54 | -54 | -11 | 12 | 4.22 |
| Superior frontal gyrus, medial, R | 13 | 53 | 19 | 8 | 4.18 |
| Middle cingulate & paracingulate gyri, R | 10 | -38 | 45 | 6 | 4.18 |
| Angular gyrus, R | 35 | -59 | 45 | 28 | 4.14 |
| Postcentral gyrus, L | -37 | -35 | 59 | 10 | 4.12 |
| Crus II of cerebellar hemisphere, L | -22 | -86 | -34 | 10 | 4.09 |
| Middle cingulate & paracingulate gyri, L | -8 | -26 | 35 | 8 | 4.06 |
| Paracentral lobule, R | 5 | -34 | 78 | 11 | 4.05 |
| Lenticular nucleus, putamen, L | -24 | -11 | 10 | 5 | 4.04 |
| IFG pars orbitalis, L | 48 | 42 | -11 | 10 | 4.02 |
| Middle frontal gyrus, L | -43 | 50 | 0 | 33 | 3.95 |
| Angular gyrus, L | -48 | -58 | 37 | 5 | 3.94 |
| Superior frontal gyrus, dorsolateral, L | -24 | 34 | 32 | 17 | 3.94 |
| Postcentral gyrus, R | 43 | -26 | 50 | 11 | 3.94 |
| Middle frontal gyrus, L | -38 | 26 | 45 | 17 | 3.93 |
| Inferior parietal gyrus, R | 48 | -46 | 45 | 9 | 3.90 |
| Lenticular nucleus, putamen, R | 35 | -13 | 0 | 6 | 3.89 |
| Paracentral lobule, L | -3 | -32 | 62 | 8 | 3.89 |
| Inferior temporal gyrus, L | -58 | -27 | -27 | 5 | 3.89 |
| Lingual gyrus, L | -29 | -93 | -14 | 6 | 3.88 |
| Superior frontal gyrus, dorsolateral, L | -22 | 32 | 42 | 6 | 3.86 |
| Crus I of cerebellar hemisphere, L | -37 | -67 | -32 | 6 | 3.85 |
| Inferior temporal gyrus, L | -58 | -30 | -22 | 9 | 3.84 |
| Posterior cingulate gyrus, R | 6 | -43 | 27 | 8 | 3.82 |
| Crus II of cerebellar hemisphere, L | -16 | -90 | -32 | 7 | 3.82 |
| Crus II of cerebellar hemisphere, L | -26 | -85 | -42 | 8 | 3.74 |
| Superior parietal gyrus, R | 29 | -59 | 58 | 5 | 3.73 |
| Inferior frontal gyrus, triangular part, L | -37 | 35 | 11 | 9 | 3.70 |
| Calcarine fissure, R | 19 | -101 | -5 | 5 | 3.70 |

^a^*K_E_*, cluster size in number of voxels; *T*, SPM T-scores; IFG, inferior frontal gyrus; L, left; R, right. Coordinates reported in MNI space. Whole-brain contrasts estimated at *P*_FDR_<0.05.
