# Supplementary material for: The Neural Basis of Avoidance Learning and Its Relation to Safety Processing
Source: eNeuro. 2026 Aug 25;13(8):ENEURO.0170-26.2026. doi: 10.1523/ENEURO.0170-26.2026 (PMC13528696; doi:10.1523/ENEURO.0170-26.2026)
Supplement: Supplementary file 2 [file eneuro-13-ENEURO.0170-26.2026-s003.docx]

**Figure 3-2**

*Differential responses to successful avoidance (D+_av_) versus unavoidable stimuli (A+_unav all_)*

|  | MNI coordinates | | |  |  |
| --- | --- | --- | --- | --- | --- |
| Regions | x | y | z | *K_E_* | *T* |
| Successful > unavoidable |  |  |  |  |  |
| Postcentral gyrus, R | 35 | -32 | 50 | 2432 | 6.93 |
| Middle occipital gyrus, L | -43 | -69 | -3 | 1161 | 6.58 |
| Posterior cingulate gyrus, R | 8 | -51 | 27 | 879 | 6.54 |
| Postcentral gyrus, L | -66 | -11 | 30 | 1919 | 6.06 |
| Middle cingulate & paracingulate gyri, R | 5 | -35 | 35 | 224 | 5.81 |
| Superior frontal gyrus, medial orbital, R | 13 | 46 | -8 | 498 | 5.55 |
| Inferior parietal gyrus, L | -29 | -66 | 43 | 453 | 5.52 |
| Superior parietal gyrus, R | 19 | -58 | 69 | 307 | 5.22 |
| Parahippocampal gyrus, R | 30 | -26 | -22 | 80 | 5.06 |
| Insula, R | 43 | -8 | 11 | 64 | 4.81 |
| Lenticular nucleus, putamen, R | 34 | -13 | -3 | 156 | 4.80 |
| Middle frontal gyrus, R | 37 | 50 | -6 | 150 | 4.76 |
| Postcentral gyrus, R | 24 | -43 | 72 | 45 | 4.70 |
| Superior frontal gyrus, dorsolateral, L | -21 | 34 | 43 | 172 | 4.69 |
| Lenticular nucleus, putamen, L | -24 | -10 | 5 | 265 | 4.65 |
| Medulla oblongata | 0 | -45 | -50 | 20 | 4.64 |
| Middle temporal gyrus, L | -61 | -14 | -24 | 42 | 4.58 |
| Lobule IV, V of cerebellar hemisphere, R | 30 | -42 | -24 | 20 | 4.58 |
| Middle temporal gyrus, R | 59 | 8 | -26 | 24 | 4.54 |
| Supplementary motor area, R | 8 | -14 | 53 | 20 | 4.52 |
| Angular gyrus, R | 51 | -62 | 29 | 130 | 4.44 |
| Fusiform gyrus, L | -30 | -40 | -18 | 15 | 4.43 |
| Gyrus rectus, R | 3 | 35 | -22 | 33 | 4.43 |
| Middle temporal gyrus, R | 58 | -54 | -3 | 88 | 4.40 |
| Superior frontal gyrus, dorsolateral, R | 24 | 34 | 45 | 24 | 4.40 |
| Lobule IV, V of cerebellar hemisphere, L | -13 | -43 | -22 | 9 | 4.40 |
| Inferior temporal gyrus, R | 53 | -13 | -27 | 21 | 4.36 |
| Middle temporal gyrus, L | -58 | 5 | -32 | 17 | 4.33 |
| Insula, L | -40 | -11 | 6 | 31 | 4.32 |
| Inferior temporal gyrus, R | 45 | -53 | -16 | 28 | 4.31 |
| Anterior cingulate cortex, supracallosal, L | -3 | -2 | 30 | 13 | 4.30 |
| Superior frontal gyrus, dorsolateral, R | 30 | 19 | 50 | 121 | 4.28 |
| Lobule VIII of cerebellar hemisphere, L | -21 | -45 | -58 | 32 | 4.27 |
| Superior parietal gyrus, L | -37 | -53 | 59 | 22 | 4.26 |
| Precuneus, R | 6 | -58 | 45 | 55 | 4.23 |
| Middle frontal gyrus, L | -38 | 16 | 54 | 90 | 4.23 |
| Superior parietal gyrus, L | -24 | -56 | 64 | 102 | 4.22 |
| Inferior temporal gyrus, L | -58 | -30 | -22 | 29 | 4.20 |
| Precuneus, L | -11 | -53 | 19 | 7 | 4.20 |
| Middle occipital gyrus, R | 40 | -70 | 5 | 90 | 4.17 |
| Middle frontal gyrus, R | 38 | 37 | 10 | 38 | 4.14 |
| Middle temporal gyrus, R | 62 | -48 | -8 | 73 | 4.12 |
| Middle frontal gyrus, L | -27 | 29 | 51 | 59 | 4.11 |
| Lenticular nucleus, putamen, R | 32 | -16 | 8 | 13 | 4.09 |
| Lobule IV, V of cerebellar hemisphere, L | -21 | -40 | -21 | 72 | 4.09 |
| Superior frontal gyrus, medial orbital, R | 11 | 29 | -13 | 10 | 4.08 |
| Temporal pole: middle temporal gyrus, R | 61 | 3 | -18 | 31 | 4.06 |
| Inferior parietal gyrus, L | -24 | -53 | 53 | 48 | 4.03 |
| Superior frontal gyrus, dorsolateral, R | 14 | 42 | 53 | 17 | 4.03 |
| Parahippocampal gyrus, R | 24 | 0 | -30 | 21 | 4.02 |
| Crus II of cerebellar hemisphere, L | -40 | -66 | -40 | 34 | 3.99 |
| Superior frontal gyrus, dorsolateral, L | -21 | 50 | 10 | 13 | 3.94 |
| Paracentral lobule, L | -3 | -32 | 61 | 12 | 3.93 |
| Temporal pole: middle temporal gyrus, L | -24 | 11 | -43 | 10 | 3.93 |
| Medial orbital gyrus, L | -21 | 35 | -24 | 8 | 3.92 |
| Anterior cingulate cortex, pregenual, L | -10 | 43 | 0 | 18 | 3.92 |
| Precentral gyrus, L | -29 | -22 | 50 | 6 | 3.91 |
| Fusiform gyrus, L | -32 | -26 | -30 | 5 | 3.89 |
| Crus I of cerebellar hemisphere, L | -56 | -58 | -26 | 7 | 3.89 |
| Lobule IX of cerebellar hemisphere, R | 14 | -51 | -58 | 8 | 3.86 |
| Angular gyrus, R | 29 | -67 | 48 | 39 | 3.85 |
| Inferior parietal gyrus, R | 58 | -43 | 46 | 5 | 3.85 |
| Middle frontal gyrus, R | 24 | 48 | 0 | 20 | 3.84 |
| Paracentral lobule, L | -11 | -24 | 78 | 9 | 3.81 |
| Inferior temporal gyrus, R | 61 | -16 | -26 | 6 | 3.80 |
| Superior parietal gyrus, L | -37 | -64 | 59 | 47 | 3.79 |
| Crus II of cerebellar hemisphere, L | -18 | -86 | -37 | 10 | 3.78 |
| Middle frontal gyrus, L | -38 | 46 | 5 | 18 | 3.77 |
| Posterior cingulate gyrus, L | -10 | -51 | 27 | 7 | 3.76 |
| Lingual gyrus, R | 11 | -45 | 3 | 8 | 3.76 |
| Hippocampus, L | -30 | -26 | -11 | 7 | 3.75 |
| Middle occipital gyrus, L | -22 | -96 | 6 | 23 | 3.75 |
| Crus I of cerebellar hemisphere, R | 40 | -69 | -37 | 24 | 3.74 |
| Middle temporal gyrus, R | 50 | -59 | -3 | 55 | 3.73 |
| Angular gyrus, R | 30 | -58 | 43 | 31 | 3.71 |
| Superior frontal gyrus, medial orbital, R | 2 | 24 | -11 | 5 | 3.71 |
| Angular gyrus, R | 40 | -56 | 29 | 7 | 3.70 |
| Lingual gyrus, L | -29 | -93 | -14 | 32 | 3.69 |
| Inferior occipital gyrus, L | -40 | -85 | -8 | 8 | 3.69 |
| Fusiform gyrus, R | 38 | -38 | -22 | 10 | 3.69 |
| Middle frontal gyrus, L | -29 | 14 | 43 | 25 | 3.68 |
| Inferior temporal gyrus, R | 54 | -40 | -14 | 9 | 3.68 |
| Temporal pole: middle temporal gyrus, L | -35 | 26 | -34 | 5 | 3.68 |
| Inferior temporal gyrus, R | 56 | -34 | -19 | 9 | 3.68 |
| Middle cingulate & paracingulate gyri, L | -8 | -27 | 35 | 5 | 3.65 |
| Middle occipital gyrus, L | -32 | -78 | 22 | 12 | 3.65 |
| Lenticular nucleus, putamen, R | 30 | -19 | 2 | 5 | 3.65 |
| Middle occipital gyrus, L | -30 | -96 | -6 | 13 | 3.64 |
| Precentral gyrus, L | -35 | -19 | 67 | 30 | 3.63 |
| Paracentral lobule, R | 5 | -30 | 66 | 6 | 3.56 |
| Middle temporal gyrus, L | -66 | -45 | -10 | 5 | 3.55 |
| Rolandic operculum, L | -40 | -6 | 16 | 6 | 3.54 |
| Superior frontal gyrus, dorsolateral, R | 18 | 32 | 38 | 8 | 3.51 |
| Superior occipital gyrus, R | 26 | -64 | 26 | 11 | 3.51 |
| Lingual gyrus, L | -13 | -99 | -14 | 13 | 3.50 |
| Middle frontal gyrus, R | 48 | 30 | 32 | 6 | 3.47 |
| Superior frontal gyrus, medial, L | -3 | 51 | 8 | 5 | 3.47 |
| Inferior temporal gyrus, R | 46 | -6 | -29 | 6 | 3.47 |
| Superior frontal gyrus, dorsolateral, R | 26 | -11 | 67 | 7 | 3.46 |
| Lobule IV, V of cerebellar hemisphere, L | -24 | -34 | -30 | 6 | 3.45 |
| Calcarine fissure, R | 22 | -101 | -5 | 5 | 3.44 |
| Supplementary motor area, R | 11 | -5 | 46 | 5 | 3.43 |
| Precentral gyrus, R | 45 | -6 | 34 | 9 | 3.41 |
| Hippocampus, L | -26 | -14 | -22 | 5 | 3.37 |
| Angular gyrus, L | -51 | -61 | 32 | 8 | 3.36 |
| Superior frontal gyrus, dorsolateral, R | 16 | 42 | 46 | 7 | 3.34 |
| Lingual gyrus, R | 22 | -85 | -14 | 5 | 3.31 |
| Superior frontal gyrus, dorsolateral, R | 19 | 29 | 56 | 5 | 3.27 |
| Middle occipital gyrus, L | -27 | -88 | 8 | 7 | 3.23 |

^a^*K_E_*, cluster size in number of voxels; *T*, SPM T-scores; L, left; R, right. Coordinates reported in MNI space. Whole-brain contrasts estimated at *P*_FDR_<0.05.
