# Supplementary material for: The Neural Basis of Avoidance Learning and Its Relation to Safety Processing
Source: eNeuro. 2026 Aug 25;13(8):ENEURO.0170-26.2026. doi: 10.1523/ENEURO.0170-26.2026 (PMC13528696; doi:10.1523/ENEURO.0170-26.2026)
Supplement: Supplementary file 3 [file eneuro-13-ENEURO.0170-26.2026-s004.docx]

**Figure 4-1**

*Avoidance learning: differential responses to successful early (D+_av early_) versus late avoidance (D+_av late_) learning and late versus early avoidance learning*

|  | MNI coordinates | | |  |  |
| --- | --- | --- | --- | --- | --- |
| Regions | x | y | z | *K_E_* | *T* |
| Early > late avoidance |  |  |  |  |  |
| Middle occipital gyrus, R | 27 | -80 | 14 | 967 | 6.05 |
| Middle occipital gyrus, L | -27 | -80 | 14 | 2131 | 5.92 |
| Precentral gyrus, L | -46 | -10 | 59 | 287 | 5.25 |
| Insula, R | 37 | 22 | -5 | 106 | 5.16 |
| Postcentral gyrus, R | 24 | -48 | 53 | 70 | 5.14 |
| Supplementary motor area, L | -10 | -5 | 75 | 25 | 5.09 |
| Lobule VI of cerebellar hemisphere, R | 32 | -59 | -22 | 569 | 5.07 |
| Supplementary motor area, R | 5 | 6 | 56 | 46 | 4.89 |
| Lobule IV, V of cerebellar hemisphere, R | 26 | -38 | -26 | 16 | 4.88 |
| Thalamus, ventral lateral, R | 13 | -16 | -2 | 28 | 4.87 |
| Lobule VI of vermis | 0 | -75 | -11 | 29 | 4.80 |
| Middle frontal gyrus, R | 42 | -2 | 58 | 100 | 4.75 |
| Periaqueductal grey | 0 | -29 | -8 | 21 | 4.69 |
| Fusiform gyrus, L | -26 | -50 | -11 | 63 | 4.67 |
| Lobule VI of cerebellar hemisphere, L | -32 | -46 | -26 | 34 | 4.62 |
| Substantia nigra, pars compacta, L | -6 | -14 | -13 | 8 | 4.55 |
| Fusiform gyrus, R | 43 | -61 | -22 | 22 | 4.52 |
| Fusiform gyrus, R | 37 | -77 | -18 | 39 | 4.51 |
| Inferior frontal gyrus, opercular part, R | 58 | 13 | 26 | 15 | 4.50 |
| Thalamus, lateral geniculate, R | 24 | -27 | -5 | 11 | 4.48 |
| Fusiform gyrus, R | 37 | -46 | -22 | 21 | 4.44 |
| Thalamus, pulvinar lateral, R | 11 | -21 | 6 | 27 | 4.40 |
| Lingual gyrus, L | -18 | -75 | -6 | 12 | 4.38 |
| Lobule VI of cerebellar hemisphere, R | 29 | -80 | -18 | 7 | 4.38 |
| Lingual gyrus, R | 14 | -85 | -10 | 74 | 4.37 |
| Precentral gyrus, L | -50 | -3 | 40 | 44 | 4.35 |
| Fusiform gyrus, R | 43 | -42 | -16 | 12 | 4.27 |
| Thalamus, mediodorsal medial, R | 0 | -13 | 3 | 21 | 4.27 |
| Lingual gyrus, R | 24 | -58 | -8 | 10 | 4.23 |
| Supplementary motor area, R | 10 | -2 | 70 | 8 | 4.08 |
| Lobule VI of vermis | 6 | -80 | -18 | 16 | 4.07 |
| Superior parietal gyrus, R | 14 | -61 | 67 | 7 | 4.03 |
| Lingual gyrus, L | -13 | -86 | -2 | 22 | 4.03 |
| Superior parietal gyrus, R | 22 | -64 | 59 | 6 | 3.94 |
| Inferior occipital gyrus, R | 37 | -77 | -8 | 16 | 3.92 |
| Precuneus, R | 10 | -77 | 51 | 10 | 3.90 |
| Supplementary motor area, R | 10 | -2 | 59 | 14 | 3.90 |
| Insula, L | -34 | 14 | 3 | 13 | 3.89 |
| Caudate nucleus, R | 10 | 2 | 8 | 10 | 3.88 |
| Inferior occipital gyrus, R | 48 | -70 | -16 | 12 | 3.88 |
| Inferior frontal gyrus, triangular part, R | 43 | 22 | 21 | 15 | 3.83 |
| Middle temporal gyrus, L | -53 | -38 | 8 | 8 | 3.82 |
| Inferior frontal gyrus, opercular part, L | -46 | 11 | 27 | 41 | 3.82 |
| Middle frontal gyrus, R | 50 | -8 | 51 | 9 | 3.80 |
| Superior occipital gyrus, R | -19 | -86 | 34 | 5 | 3.78 |
| Fusiform gyrus, R | 27 | -32 | -18 | 9 | 3.77 |
| Inferior temporal gyrus, L | -42 | -48 | -19 | 6 | 3.76 |
| Insula, R | 34 | 16 | 8 | 5 | 3.70 |
| Lingual gyrus, R | 22 | -82 | -6 | 7 | 3.70 |
| Inferior occipital gyrus, R | 45 | -75 | -13 | 6 | 3.69 |
| Superior parietal gyrus, L | -22 | -62 | 62 | 6 | 3.64 |
| Lobule VIII of vermis | -2 | -72 | -40 | 5 | 3.50 |
| Late > early avoidance |  |  |  |  |  |
| Paracentral lobule, L | -13 | -35 | 72 | 20 | 5.67 |
| Lobule VI of cerebellar hemisphere, R | 30 | -67 | -29 | 12 | 5.48 |
| Lobule VI of cerebellar hemisphere, R | 21 | -67 | -29 | 13 | 5.22 |
| Middle frontal gyrus, L | -38 | 26 | 42 | 22 | 5.08 |
| Inferior parietal gyrus, R | 48 | -42 | 54 | 5 | 4.91 |
| Paracentral lobule, L | -5 | -35 | 56 | 7 | 4.81 |

^a^*K_E_*, cluster size in number of voxels; *T*, SPM T-scores; L, left; R, right. Coordinates reported in MNI space. Whole-brain contrasts estimated at *P*_FDR_<0.05.
