# Supplementary material for: The Neural Basis of Avoidance Learning and Its Relation to Safety Processing
Source: eNeuro. 2026 Aug 25;13(8):ENEURO.0170-26.2026. doi: 10.1523/ENEURO.0170-26.2026 (PMC13528696; doi:10.1523/ENEURO.0170-26.2026)
Supplement: Supplementary file 5 [file eneuro-13-ENEURO.0170-26.2026-s007.docx]

**Figure 5-3**

*Differential responses to successful avoidance (D+_av_) versus safety learning (AX-_cond_) masked by the results of a conjunction-null analysis of D+_av_ and AX-_cond_*

|  | MNI coordinates | | |  |  |
| --- | --- | --- | --- | --- | --- |
| Regions | x | y | z | *K_E_* | *T* |
| Successful avoidance > safety learning (masked with conjunction) |  |  |  |  |  |
| Crus I of cerebellar hemisphere, R | 19 | -69 | -34 | 10026 | 7.82 |
| Middle cingulate & paracingulate gyri, R | 5 | -30 | 34 | 9807 | 7.48 |
| Superior frontal gyrus, medial orbital, R | 3 | 45 | -8 | 4919 | 6.87 |
| Superior temporal gyrus, R | 56 | 0 | -13 | 1366 | 5.77 |
| Superior temporal gyrus, L | -45 | 0 | -13 | 1162 | 5.61 |
| Supramarginal gyrus, L | -64 | -42 | 34 | 632 | 5.58 |
| Medulla oblongata,R | 2 | -45 | -50 | 503 | 5.46 |
| Postcentral gyrus, R | 53 | -5 | 27 | 368 | 5.42 |
| Inferior frontal gyrus, triangular part, L | -48 | 32 | 13 | 1047 | 5.41 |
| Inferior temporal gyrus, L | -34 | -5 | -43 | 43 | 5.17 |
| Postcentral gyrus, L | -42 | -18 | 32 | 1004 | 5.06 |
| Inferior frontal gyrus, triangular part, L | 42 | 22 | 19 | 57 | 4.86 |
| Middle frontal gyrus, L | -29 | 11 | 54 | 109 | 4.8 |
| SupraMarginal gyrus, R | 56 | -35 | 37 | 1720 | 4.79 |
| Inferior frontal gyrus, triangular part, R | 46 | 37 | 8 | 200 | 4.65 |
| Crus I of cerebellar hemisphere, R | 53 | -48 | -38 | 34 | 4.58 |
| Superior frontal gyrus, dorsolateral, R | 21 | 21 | 35 | 105 | 4.57 |
| Lobule I, II of vermis | 3 | -40 | -26 | 162 | 4.52 |
| Hippocampus, L | -19 | -42 | 3 | 165 | 4.5 |
| Superior frontal gyrus, dorsolateral, R | 27 | 45 | 43 | 532 | 4.42 |
| Temporal pole: superior temporal gyrus, L | -35 | 22 | -32 | 50 | 4.4 |
| Olfactory cortex, R | 10 | 10 | -18 | 29 | 4.32 |
| Inferior temporal gyrus, L | -42 | -26 | -21 | 64 | 4.26 |
| Angular gyrus, R | 35 | -59 | 43 | 81 | 4.24 |
| Middle occipital gyrus, R | 26 | -91 | 0 | 475 | 4.23 |
| Temporal pole: middle temporal gyrus, R | 38 | 26 | -35 | 41 | 4.21 |
| Inferior temporal gyrus, L | -32 | 8 | -42 | 64 | 4.18 |
| Middle temporal gyrus, R | 61 | -45 | -3 | 38 | 4.11 |
| Middle cingulate & paracingulate gyri, L | -6 | -5 | 29 | 125 | 4.07 |
| Fusiform gyrus, R | 42 | -40 | -16 | 58 | 4.04 |
| Thalamus, pulvinar medial, L | -16 | -30 | 13 | 48 | 3.97 |
| Fusiform gyrus, R | 30 | -13 | -32 | 23 | 3.93 |
| Middle temporal gyrus, L | -54 | -50 | 18 | 19 | 3.92 |
| Hippocampus, R | 19 | -34 | 2 | 13 | 3.9 |
| Inferior temporal gyrus, R | 58 | -61 | -19 | 6 | 3.88 |
| Inferior temporal gyrus, L | -62 | -46 | -22 | 5 | 3.87 |
| Middle cingulate & paracingulate gyri, L | -13 | -13 | 40 | 15 | 3.85 |
| Middle occipital gyrus, L | -29 | -99 | 3 | 84 | 3.84 |
| Middle temporal gyrus, R | 53 | -37 | -8 | 27 | 3.8 |
| Caudate nucleus, R | 16 | 24 | 13 | 28 | 3.78 |
| Hippocampus, R | 16 | -10 | -16 | 28 | 3.78 |
| Middle frontal gyrus, R | -34 | 27 | 51 | 73 | 3.78 |
| Thalamus, ventral lateral, R | 16 | -13 | 14 | 20 | 3.77 |
| Middle frontal gyrus, R | 37 | 2 | 43 | 19 | 3.76 |
| Lobule IV, V of cerebellar hemisphere, R | 26 | -27 | -30 | 90 | 3.73 |
| Angular gyrus, L | -43 | -50 | 27 | 42 | 3.73 |
| Lobule III of cerebellar hemisphere, R | 13 | -35 | -22 | 24 | 3.73 |
| Nucleus accumbens, R | 6 | 6 | -10 | 15 | 3.73 |
| Inferior parietal gyrus, R | 45 | -54 | 54 | 35 | 3.7 |
| Rolandic operculum, L | -37 | 3 | 14 | 28 | 3.69 |
| Inferior temporal gyrus, L | -56 | -8 | -40 | 12 | 3.68 |
| Inferior temporal gyrus, R | 46 | -16 | -26 | 17 | 3.67 |
| Precuneus, L | -13 | -53 | 50 | 14 | 3.64 |
| Crus I of cerebellar hemisphere, R | 35 | -78 | -32 | 13 | 3.6 |
| Supplementary motor area, L | -10 | -3 | 45 | 6 | 3.59 |
| Lobule III of cerebellar hemisphere, L | -10 | -35 | -22 | 18 | 3.58 |
| Lobule VIIB of cerebellar hemisphere, R | 43 | -40 | -45 | 20 | 3.57 |
| Gyrus rectus, R | 5 | 19 | -26 | 7 | 3.56 |
| Superior temporal gyrus, R | 51 | -18 | -8 | 29 | 3.53 |
| Superior temporal gyrus, L | -50 | -22 | 2 | 5 | 3.53 |
| Middle temporal gyrus, L | -61 | -13 | -8 | 35 | 3.52 |
| Substantia nigra, pars reticulata, L | -8 | -19 | -19 | 6 | 3.52 |
| Temporal pole: superior temporal gyrus, L | -26 | 16 | -34 | 16 | 3.52 |
| Superior frontal gyrus, dorsolateral, L | -16 | 22 | 46 | 52 | 3.51 |
| Crus I of cerebellar hemisphere, R | 46 | -43 | -34 | 53 | 3.51 |
| Lobule IX of cerebellar hemisphere, L | -13 | -48 | -42 | 9 | 3.49 |
| Middle temporal gyrus, L | -56 | -14 | -22 | 9 | 3.48 |
| Superior frontal gyrus, dorsolateral, R | 14 | 66 | 19 | 26 | 3.46 |
| Thalamus, ventral lateral, R | 11 | -6 | 6 | 17 | 3.44 |
| Crus I of cerebellar hemisphere, R | 37 | -78 | -26 | 17 | 3.43 |
| Middle temporal gyrus, L | -51 | -19 | -18 | 5 | 3.43 |
| Inferior frontal gyrus, triangular part, L | -48 | 22 | 18 | 29 | 3.42 |
| Hippocampus, R | 38 | -16 | -19 | 13 | 3.42 |
| Postcentral gyrus, R | 16 | -35 | 59 | 6 | 3.41 |
| Inferior parietal gyrus, L | -53 | -46 | 54 | 11 | 3.41 |
| Superior frontal gyrus, dorsolateral, L | -18 | 67 | -11 | 25 | 3.41 |
| Inferior temporal gyrus, L | -51 | -34 | -19 | 5 | 3.41 |
| Superior temporal gyrus, R | 51 | -6 | -3 | 12 | 3.4 |
| Fusiform gyrus, R | 35 | -51 | -10 | 6 | 3.39 |
| Superior temporal gyrus, R | 64 | -30 | 2 | 25 | 3.39 |
| Lobule VIII of cerebellar hemisphere, L | -22 | -42 | -54 | 8 | 3.39 |
| Medial orbital gyrus, R | 18 | 29 | -21 | 7 | 3.38 |
| Posterior cingulate gyrus, L | -8 | -45 | 22 | 6 | 3.37 |
| Middle temporal gyrus, L | -51 | -27 | -16 | 6 | 3.36 |
| Caudate nucleus, L | -16 | 8 | 24 | 17 | 3.35 |
| Pons | -10 | -35 | -43 | 12 | 3.34 |
| Posterior orbital gyrus, R | 32 | 30 | -16 | 9 | 3.33 |
| Superior frontal gyrus, dorsolateral, L | -21 | 61 | 6 | 8 | 3.33 |
| Inferior temporal gyrus, L | -59 | -46 | -27 | 16 | 3.33 |
| Superior frontal gyrus, dorsolateral, L | -16 | 61 | 10 | 14 | 3.32 |
| Caudate nucleus, R | 8 | 6 | 8 | 14 | 3.3 |
| Precuneus, R | 2 | -67 | 62 | 9 | 3.28 |
| Middle frontal gyrus, L | -30 | 16 | 45 | 24 | 3.27 |
| Superior frontal gyrus, medial, L | 0 | 64 | 0 | 11 | 3.26 |
| Superior frontal gyrus, dorsolateral, R | 21 | 56 | -3 | 19 | 3.26 |
| Middle temporal gyrus, L | -43 | -59 | 18 | 15 | 3.25 |
| Middle occipital gyrus, L | -34 | -86 | 34 | 5 | 3.25 |
| Lenticular nucleus, putamen, L | -24 | 19 | 3 | 7 | 3.24 |
| Pons | 5 | -29 | -38 | 7 | 3.23 |
| Supplementary motor area, R | 10 | -21 | 62 | 27 | 3.22 |
| Amygdala, L | -22 | 0 | -18 | 30 | 3.22 |
| Medial orbital gyrus, R | 6 | 53 | -26 | 5 | 3.2 |
| Lobule VIII of cerebellar hemisphere, R | 26 | -42 | -56 | 9 | 3.2 |
| Superior frontal gyrus, medial, R | 10 | 61 | 13 | 56 | 3.2 |
| Lobule IV, V of cerebellar hemisphere, R | 22 | -30 | -24 | 9 | 3.17 |
| Crus II of cerebellar hemisphere, L | -26 | -83 | -37 | 14 | 3.16 |
| Inferior frontal gyrus, triangular part, L | -34 | 24 | 11 | 7 | 3.16 |
| Lenticular nucleus, putamen, L | -29 | -2 | 2 | 7 | 3.16 |
| Middle frontal gyrus, R | 40 | 43 | 6 | 5 | 3.15 |
| Inferior parietal gyrus, L | -53 | -51 | 51 | 5 | 3.14 |
| Angular gyrus, L | -50 | -53 | 34 | 14 | 3.13 |
| Middle temporal gyrus, L | -54 | -37 | 8 | 11 | 3.11 |
| Crus I of cerebellar hemisphere, L | -26 | -83 | -24 | 5 | 3.1 |
| Middle temporal gyrus, L | -69 | -27 | -5 | 6 | 3.09 |
| Superior temporal gyrus, L | -59 | -10 | 2 | 5 | 3.08 |
| Superior frontal gyrus, medial, R | 2 | 62 | 21 | 9 | 3.06 |
| Superior frontal gyrus, dorsolateral, L | -21 | 18 | 54 | 9 | 3.06 |
| Thalamus, ventral posterolateral, L | -19 | -21 | 11 | 5 | 3.04 |
| Superior frontal gyrus, dorsolateral, R | 21 | 53 | 5 | 7 | 3.04 |
| Calcarine fissure, R | 26 | -70 | 6 | 8 | 3.03 |
| Thalamus, pulvinar medial, R | 3 | -24 | 5 | 6 | 3.02 |
| Hippocampus, R | 38 | -26 | -14 | 6 | 2.95 |
| Middle frontal gyrus, L | -34 | 29 | 45 | 9 | 2.95 |
| Middle frontal gyrus, R | 38 | 53 | 19 | 7 | 2.93 |
| Middle frontal gyrus, L | -27 | 43 | 24 | 5 | 2.92 |
| Middle frontal gyrus, L | -45 | 21 | 34 | 6 | 2.89 |

^a^*K_E_*, cluster size in number of voxels; *T*, SPM T-scores; L, left; R, right. Coordinates reported in MNI space. Whole-brain contrasts estimated at *P*_FDR_<0.05.
