# Supplementary material for: The Neural Basis of Avoidance Learning and Its Relation to Safety Processing
Source: eNeuro. 2026 Aug 25;13(8):ENEURO.0170-26.2026. doi: 10.1523/ENEURO.0170-26.2026 (PMC13528696; doi:10.1523/ENEURO.0170-26.2026)
Supplement: Supplementary file 6 [file eneuro-13-ENEURO.0170-26.2026-s009.docx]

**Figure 5-5**

*Differential responses to late successful avoidance trials (D+_av late_) versus late safety learning trials (AX-_cond late_)*

|  | MNI coordinates | | |  |  |
| --- | --- | --- | --- | --- | --- |
| Regions | x | y | z | *K_E_* | *T* |
| Late successful avoidance > late safety learning |  |  |  |  |  |
| Middle cingulate & paracingulate gyri, R | 8 | -35 | 27 | 6165 | 7.46 |
| Lobule VI of cerebellar hemisphere, R | 21 | -66 | -32 | 3228 | 7.05 |
| Middle temporal gyrus, L | -64 | -50 | -2 | 4540 | 6.57 |
| Angular gyrus, L | -43 | -66 | 42 | 4849 | 6.17 |
| Superior frontal gyrus, medial orbital, R | 3 | 53 | -3 | 4194 | 5.99 |
| Middle frontal gyrus, L | -32 | 32 | 45 | 2833 | 5.46 |
| Angular gyrus, R | 51 | -59 | 34 | 2433 | 5.44 |
| Lobule IX of cerebellar hemisphere, R | 8 | -48 | -46 | 212 | 5.44 |
| Inferior temporal gyrus, L | -56 | -61 | -26 | 77 | 5.33 |
| Middle temporal gyrus, L | -54 | -10 | -13 | 470 | 5.23 |
| Insula, R | 29 | 21 | -18 | 102 | 5.16 |
| Temporal pole: superior temporal gyrus, R | 62 | 5 | -6 | 452 | 5.14 |
| Inferior frontal gyrus, triangular part, L | -48 | 32 | 11 | 679 | 5.1 |
| Postcentral gyrus, L | -22 | -40 | 78 | 358 | 4.72 |
| Superior frontal gyrus, dorsolateral, L | -18 | 61 | 26 | 83 | 4.66 |
| Middle temporal gyrus, L | -51 | 0 | -24 | 340 | 4.62 |
| Middle temporal gyrus, R | 62 | -45 | -3 | 178 | 4.54 |
| Postcentral gyrus, R | 18 | -34 | 72 | 249 | 4.47 |
| Middle temporal gyrus, R | 53 | -37 | -8 | 52 | 4.35 |
| Hippocampus, L | -30 | -24 | -13 | 32 | 4.31 |
| Postcentral gyrus, R | 42 | -14 | 34 | 105 | 4.28 |
| Caudate nucleus, L | -10 | 14 | -3 | 35 | 4.27 |
| Superior temporal gyrus, R | 58 | -11 | -11 | 61 | 4.26 |
| Inferior temporal gyrus, L | -45 | -29 | -26 | 68 | 4.23 |
| Inferior temporal gyrus, R | 56 | -50 | -18 | 25 | 4.22 |
| Inferior temporal gyrus, L | -59 | -16 | -26 | 145 | 4.19 |
| Temporal pole: superior temporal gyrus, R | 45 | 24 | -24 | 32 | 4.19 |
| Middle temporal gyrus, R | 59 | -54 | 2 | 53 | 4.18 |
| Temporal pole: superior temporal gyrus, L | -32 | 6 | -21 | 32 | 4.15 |
| Parahippocampal gyrus, R | 22 | 2 | -32 | 52 | 4.1 |
| Parahippocampal gyrus, L | -22 | 3 | -27 | 111 | 4.09 |
| Thalamus, ventral anterior, R | 14 | -3 | 10 | 12 | 4 |
| Hippocampus, R | 34 | -6 | -22 | 39 | 4 |
| Middle frontal gyrus, L | -35 | 29 | 27 | 27 | 3.98 |
| Hippocampus, R | 27 | -10 | -19 | 23 | 3.95 |
| Inferior occipital gyrus, R | 24 | -96 | -2 | 122 | 3.95 |
| Nucleus accumbens, R | 5 | 16 | -2 | 37 | 3.92 |
| Temporal pole: superior temporal gyrus, L | -48 | 16 | -18 | 32 | 3.92 |
| Olfactory cortex, L | 0 | 11 | -14 | 15 | 3.92 |
| Temporal pole: middle temporal gyrus, L | -29 | 6 | -43 | 51 | 3.91 |
| Lenticular nucleus, putamen, L | -32 | -13 | -10 | 24 | 3.85 |
| Superior frontal gyrus, medial, L | -2 | 62 | 30 | 30 | 3.85 |
| Temporal pole: middle temporal gyrus, L | -34 | 21 | -34 | 75 | 3.84 |
| Temporal pole: middle temporal gyrus, R | 40 | 26 | -34 | 27 | 3.84 |
| Hippocampus, L | -34 | -14 | -19 | 49 | 3.83 |
| Temporal pole: superior temporal gyrus, R | 35 | 6 | -22 | 26 | 3.83 |
| Lobule IV, V of cerebellar hemisphere, L | -10 | -38 | -16 | 15 | 3.83 |
| Crus II of cerebellar hemisphere, R | 51 | -45 | -42 | 18 | 3.8 |
| Inferior temporal gyrus, L | -53 | -34 | -19 | 22 | 3.79 |
| Inferior frontal gyrus, opercular part, L | -38 | 5 | 16 | 15 | 3.78 |
| Superior temporal gyrus, L | -62 | -19 | 6 | 6 | 3.77 |
| Precuneus, L | -13 | -53 | 50 | 9 | 3.77 |
| Superior frontal gyrus, dorsolateral, R | 24 | 34 | 42 | 116 | 3.75 |
| Olfactory cortex, R | 11 | 8 | -16 | 13 | 3.74 |
| Lobule IV, V of cerebellar hemisphere, L | -21 | -29 | -26 | 10 | 3.73 |
| Nucleus accumbens, R | 8 | 10 | -8 | 12 | 3.73 |
| Caudate nucleus, L | -18 | 8 | 24 | 29 | 3.72 |
| Middle temporal gyrus, L | -58 | -35 | 6 | 15 | 3.72 |
| Inferior frontal gyrus, triangular part, R | 53 | 38 | 6 | 69 | 3.69 |
| Superior frontal gyrus, medial, R | 11 | 34 | 62 | 43 | 3.69 |
| Gyrus rectus, R | 11 | 21 | -16 | 13 | 3.68 |
| Superior frontal gyrus, medial, L | -10 | 35 | 32 | 8 | 3.67 |
| Postcentral gyrus, L | -42 | -18 | 32 | 44 | 3.66 |
| Middle frontal gyrus, R | 40 | 46 | 34 | 99 | 3.64 |
| Superior frontal gyrus, dorsolateral, R | 22 | 21 | 64 | 36 | 3.63 |
| Temporal pole: middle temporal gyrus, R | 34 | 16 | -37 | 15 | 3.63 |
| Superior temporal gyrus, L | -67 | -18 | 2 | 5 | 3.61 |
| Superior frontal gyrus, dorsolateral, R | 29 | 45 | 45 | 38 | 3.61 |
| Caudate nucleus, L | -13 | 24 | -5 | 13 | 3.6 |
| Superior frontal gyrus, dorsolateral, R | 21 | 56 | -2 | 17 | 3.57 |
| Hippocampus, R | 35 | -19 | -13 | 8 | 3.53 |
| Middle frontal gyrus, L | -42 | 14 | 42 | 12 | 3.52 |
| Superior frontal gyrus, dorsolateral, L | -24 | 46 | -6 | 11 | 3.52 |
| Middle temporal gyrus, L | -34 | 18 | -43 | 11 | 3.52 |
| Superior frontal gyrus, medial, R | 8 | 64 | 29 | 29 | 3.51 |
| Precuneus, L | -2 | -53 | 69 | 77 | 3.5 |
| Postcentral gyrus, L | -54 | -11 | 26 | 41 | 3.49 |
| Inferior temporal gyrus, L | -54 | -8 | -40 | 23 | 3.49 |
| Olfactory cortex, L | -2 | 19 | -19 | 17 | 3.46 |
| Rolandic operculum, L | -42 | -16 | 22 | 5 | 3.46 |
| Inferior temporal gyrus, L | -56 | -40 | -22 | 8 | 3.46 |
| Olfactory cortex, L | -6 | 10 | -14 | 6 | 3.46 |
| Rolandic operculum, R | 51 | -6 | 14 | 5 | 3.45 |
| Middle occipital gyrus, L | -29 | -99 | 2 | 16 | 3.44 |
| Amygdala, R | 32 | -2 | -11 | 6 | 3.44 |
| Inferior frontal gyrus, triangular part, R | 40 | 34 | 6 | 12 | 3.43 |
| Superior frontal gyrus, dorsolateral, R | 16 | 45 | 54 | 27 | 3.43 |
| Superior frontal gyrus, dorsolateral, L | -10 | 62 | 35 | 16 | 3.41 |
| Inferior frontal gyrus, triangular part, L | -48 | 37 | 26 | 24 | 3.4 |
| Superior frontal gyrus, medial, L | -2 | 30 | 58 | 15 | 3.4 |
| Lenticular nucleus, putamen, L | -27 | -13 | 10 | 9 | 3.39 |
| Hippocampus, R | 34 | -27 | -8 | 15 | 3.38 |
| Superior frontal gyrus, medial, L | -2 | 43 | 53 | 15 | 3.38 |
| IFG pars orbitalis, L | -53 | 37 | -11 | 12 | 3.37 |
| Crus II of cerebellar hemisphere, L | -6 | -88 | -22 | 15 | 3.37 |
| Hippocampus, R | 19 | -34 | 3 | 5 | 3.37 |
| Inferior temporal gyrus, L | -59 | -43 | -22 | 9 | 3.36 |
| Lobule III of cerebellar hemisphere, R | 13 | -35 | -22 | 22 | 3.35 |
| Anterior cingulate cortex, supracallosal, L | -2 | 18 | 26 | 19 | 3.35 |
| Medial orbital gyrus, L | -14 | 45 | -19 | 8 | 3.33 |
| Superior frontal gyrus, dorsolateral, R | 14 | 67 | 21 | 10 | 3.31 |
| Parahippocampal gyrus, L | -27 | -40 | -8 | 5 | 3.31 |
| Superior frontal gyrus, medial, L | -3 | 51 | 40 | 7 | 3.31 |
| Superior frontal gyrus, dorsolateral, R | 14 | 16 | 50 | 6 | 3.3 |
| Superior occipital gyrus, R | 16 | -98 | 18 | 20 | 3.3 |
| Middle frontal gyrus, L | -38 | 40 | 30 | 8 | 3.29 |
| Superior frontal gyrus, dorsolateral, R | 30 | 54 | 18 | 8 | 3.26 |
| Crus I of cerebellar hemisphere, R | 50 | -53 | -38 | 6 | 3.25 |
| Crus I of cerebellar hemisphere, R | 42 | -50 | -35 | 34 | 3.25 |
| Inferior parietal gyrus, R | 40 | -48 | 37 | 13 | 3.25 |
| Paracentral lobule, R | 5 | -45 | 80 | 9 | 3.24 |
| Middle temporal gyrus, R | 58 | -5 | -27 | 7 | 3.23 |
| Inferior temporal gyrus, L | -35 | -5 | -45 | 6 | 3.21 |
| Amygdala, L | -22 | -2 | -18 | 8 | 3.21 |
| Inferior occipital gyrus, L | -21 | -99 | -13 | 5 | 3.19 |
| Middle occipital gyrus, L | -24 | -98 | -5 | 16 | 3.19 |
| Postcentral gyrus, R | 51 | -6 | 27 | 16 | 3.17 |
| Lobule VIII of cerebellar hemisphere, R | 30 | -43 | -48 | 15 | 3.16 |
| Postcentral gyrus, R | 14 | -42 | 64 | 6 | 3.15 |
| Inferior parietal gyrus, L | -46 | -48 | 59 | 5 | 3.15 |
| Gyrus rectus, L | -11 | 27 | -19 | 12 | 3.14 |
| Calcarine fissure and surrounding cortex, L | -14 | -104 | -2 | 19 | 3.14 |
| Superior frontal gyrus, dorsolateral, R | 19 | 29 | 38 | 12 | 3.14 |
| Superior frontal gyrus, dorsolateral, R | 21 | 53 | 5 | 6 | 3.12 |
| Lobule VIII of cerebellar hemisphere, R | 29 | -61 | -54 | 12 | 3.12 |
| Lingual gyrus, L | -13 | -98 | -18 | 7 | 3.11 |
| Paracentral lobule, R | 5 | -35 | 80 | 10 | 3.11 |
| Lobule VIII of cerebellar hemisphere, L | -22 | -61 | -48 | 6 | 3.10 |
| Superior frontal gyrus, dorsolateral, L | -27 | 61 | 0 | 10 | 3.10 |
| Parahippocampal gyrus, R | 34 | -26 | -18 | 7 | 3.09 |
| Precuneus, L | -3 | -77 | 50 | 28 | 3.09 |
| Lobule VI of cerebellar hemisphere, R | 43 | -38 | -32 | 5 | 3.06 |
| Gyrus rectus, L | -14 | 19 | -11 | 5 | 3.06 |
| Middle temporal gyrus, L | -51 | -27 | -16 | 8 | 3.06 |
| Middle temporal gyrus, R | 61 | -48 | 8 | 6 | 3.05 |
| Lobule VIII of cerebellar hemisphere, L | -35 | -37 | -43 | 5 | 3.05 |
| Anterior cingulate cortex, supracallosal, L | -11 | 32 | 27 | 9 | 3.04 |
| Superior frontal gyrus, medial orbital, R | 6 | 66 | -16 | 10 | 3.03 |
| Middle frontal gyrus, L | -48 | 13 | 46 | 6 | 3.03 |
| Insula, L | -35 | 5 | -14 | 6 | 3.01 |
| Anterior cingulate cortex, pregenual, L | -5 | 42 | 3 | 13 | 2.97 |
| Middle frontal gyrus, R | 43 | 16 | 50 | 5 | 2.90 |
| Hippocampus, R | 27 | -37 | 3 | 6 | 2.88 |

^a^*K_E_*, cluster size in number of voxels; *T*, SPM T-scores; IFG, inferior frontal gyrus; L, left; R, right. Coordinates reported in MNI space. Whole-brain contrasts estimated at *P*_FDR_<0.05.
