# Supplementary material for: The Neural Basis of Avoidance Learning and Its Relation to Safety Processing
Source: eNeuro. 2026 Aug 25;13(8):ENEURO.0170-26.2026. doi: 10.1523/ENEURO.0170-26.2026 (PMC13528696; doi:10.1523/ENEURO.0170-26.2026)
Supplement: Supplementary file 7 [file eneuro-13-ENEURO.0170-26.2026-s010.docx]

**Figure 5-6**

*Differential responses to safety learning (AX-_cond_) versus successful avoidance (D+_av_)*

|  | MNI coordinates | | |  |  |
| --- | --- | --- | --- | --- | --- |
| Regions | x | y | z | *K_E_* | *T* |
| Safety learning > successful avoidance |  |  |  |  |  |
| Lingual gyrus, L | -13 | -74 | -11 | 28232 | 9.24 |
| Superior parietal gyrus, L | -18 | -61 | 62 | 761 | 6.83 |
| Inferior frontal gyrus, opercular part, R | 56 | 14 | 21 | 978 | 5.82 |
| IFG pars orbitalis, L | -40 | 27 | -6 | 333 | 5.73 |
| Middle temporal gyrus, L | -46 | -66 | 8 | 669 | 5.34 |
| Middle temporal gyrus, R | 45 | -62 | 6 | 642 | 5.25 |
| Medulla oblongata, R | -40 | -37 | 19 | 257 | 4.78 |
| Middle cingulate & paracingulate gyri, L | -8 | -21 | 46 | 73 | 4.78 |
| Thalamus, intralaminar, L | -8 | -18 | -2 | 183 | 4.74 |
| Lobule VI of vermis | 3 | -64 | -22 | 56 | 4.71 |
| Thalamus, lateral geniculate, R | 24 | -22 | -6 | 38 | 4.60 |
| Middle cingulate & paracingulate gyri, R | 11 | -10 | 48 | 101 | 4.40 |
| Insula, R | 42 | -2 | 5 | 111 | 4.28 |
| Insula, L | -34 | 18 | -11 | 30 | 4.25 |
| Hippocampus, L | -21 | -24 | -8 | 39 | 4.23 |
| Rolandic operculum, R | 46 | -22 | 19 | 67 | 4.11 |
| Postcentral gyrus, L | -58 | -22 | 29 | 22 | 3.98 |
| Lobule IV, V of cerebellar hemisphere, R | 8 | -53 | -11 | 17 | 3.93 |
| Lobule IX of cerebellar hemisphere, L | -13 | -48 | -53 | 29 | 3.93 |
| Insula, R | 30 | 16 | -11 | 44 | 3.92 |
| Lenticular nucleus, pallidum, L | -19 | -6 | -8 | 28 | 3.84 |
| Substantia nigra, pars compacta, R | 11 | -18 | -8 | 24 | 3.82 |
| Temporal pole: middle temporal gyrus, L | -53 | 5 | -37 | 8 | 3.79 |
| Pons | 2 | -22 | -42 | 6 | 3.76 |
| Postcentral gyrus, L | -35 | -34 | 40 | 32 | 3.75 |
| Superior frontal gyrus, medial, R | 8 | 46 | 40 | 42 | 3.66 |
| Lobule IX of vermis | 2 | -53 | -40 | 26 | 3.57 |
| Superior frontal gyrus, dorsolateral, L | -13 | 53 | 37 | 51 | 3.57 |
| Middle occipital gyrus, R | 34 | -86 | 26 | 15 | 3.56 |
| Superior frontal gyrus, medial, R | 8 | 29 | 38 | 9 | 3.53 |
| Thalamus, pulvinar medial, L | -13 | -27 | 6 | 6 | 3.50 |
| Inferior frontal gyrus, opercular part, L | -50 | 14 | 3 | 14 | 3.50 |
| Superior frontal gyrus, medial, L | -6 | 42 | 42 | 40 | 3.43 |
| Middle frontal gyrus, L | -42 | 54 | -11 | 7 | 3.42 |
| Thalamus, ventral lateral, L | -14 | -10 | -2 | 6 | 3.41 |
| Thalamus, ventral posterolateral, R | 14 | -19 | 3 | 13 | 3.37 |
| Middle temporal gyrus, L | -46 | -38 | 3 | 11 | 3.35 |
| Caudate nucleus, L | -16 | 6 | 13 | 8 | 3.35 |
| Anterior orbital gyrus, R | 32 | 45 | -16 | 8 | 3.33 |
| Rolandic operculum, L | -43 | -8 | 13 | 9 | 3.33 |
| Fusiform gyrus, R | 34 | -72 | -14 | 15 | 3.32 |
| Caudate nucleus, R | 18 | 11 | 16 | 8 | 3.29 |
| Inferior temporal gyrus, R | 43 | -66 | -6 | 12 | 3.28 |
| Rolandic operculum, L | -58 | -2 | 8 | 6 | 3.28 |
| Inferior temporal gyrus, L | -43 | 10 | -42 | 5 | 3.26 |
| Inferior frontal gyrus, triangular part, L | -58 | 21 | 8 | 13 | 3.21 |
| Medial orbital gyrus, R | 14 | 19 | -27 | 5 | 3.20 |
| Lobule X of cerebellar hemisphere, L | -21 | -40 | -43 | 5 | 3.18 |
| Lobule VIII of vermis | 5 | -67 | -30 | 6 | 3.16 |
| Middle cingulate & paracingulate gyri, L | 0 | -21 | 48 | 9 | 3.11 |
| IFG pars orbitalis, R | 43 | 38 | -11 | 15 | 3.10 |
| Thalamus, pulvinar lateral, L | -8 | -22 | 8 | 6 | 3.01 |
| Paracentral lobule, R | 14 | -43 | 53 | 10 | 3.00 |

^a^*K_E_*, cluster size in number of voxels; *T*, SPM T-scores; IFG, inferior frontal gyrus; L, left; R, right. Coordinates reported in MNI space. Whole-brain contrasts estimated at *P*_FDR_<0.05.
